# An Investigation on Formaldehyde Emission Characteristics of Wood Building Materials in Chinese Standard Tests: Product Emission Levels, Measurement Uncertainties, and Data Correlations between Various Tests

**DOI:** 10.1371/journal.pone.0144374

**Published:** 2015-12-10

**Authors:** Wei Song, Yang Cao, Dandan Wang, Guojun Hou, Zaihua Shen, Shuangbao Zhang

**Affiliations:** 1 Beijing Key Laboratory of Wood Science and Engineering, Beijing Forestry University, Beijing, China; 2 MOE Key Laboratory of Wooden Material Science and Application, Beijing Forestry University, Beijing, China; 3 MOE Engineering Research Center of Forestry Biomass Materials and Bioenergy, Beijing Forestry University, Beijing, China; 4 R & D Center for Natural Fiber Composites and Environmentally Friendly Adhesives, Zhejiang Chengzhu Advanced Material Technology Co., Ltd., Shaoxing, China; Tsinghua University, CHINA

## Abstract

As a large producer and consumer of wood building materials, China suffers product formaldehyde emissions (PFE) but lacks systematic investigations and basic data on Chinese standard emission tests (CST), so this paper presented a first effort on this issue. The PFE of fiberboards, particleboards, blockboards, floorings, and parquets manufactured in Beijing region were characterized by the perforator extraction method (PE), 9–11 L and 40 L desiccator methods (D9, D40), and environmental chamber method (EC) of the Chinese national standard GB 18580; based on statistics of PFE data, measurement uncertainties in CST were evaluated by the Monte Carlo method; moreover, PFE data correlations between tests were established. Results showed: (1) Different tests may give slightly different evaluations on product quality. In PE and D9 tests, blockboards and parquets reached E1 grade for PFE, which can be directly used in indoor environment; but in D40 and EC tests, floorings and parquets achieved E1. (2) In multiple tests, PFE data characterized by PE, D9, and D40 complied with Gaussian distributions, while those characterized by EC followed log-normal distributions. Uncertainties in CST were overall low, with uncertainties for 20 material-method combinations all below 7.5%, and the average uncertainty for each method under 3.5%, thus being acceptable in engineering application. A more complicated material structure and a larger test scale caused higher uncertainties. (3) Conventional linear models applied to correlating PFE values between PE, D9, and EC, with R^2^ all over 0.840, while novel logarithmic (exponential) models can work better for correlations involving D40, with R^2^ all beyond 0.901. This research preliminarily demonstrated the effectiveness of CST, where results for D40 presented greater similarities to EC—the currently most reliable test for PFE, thus highlighting the potential of Chinese D40 as a more practical approach in production control and risk assessment.

## Introduction

Around the globe, people spend approximately 90% of their time indoors, thus making the indoor air quality have a significant impact on the modern life [1]. However, volatile organic contaminants like the formaldehyde emitted from a wide range of building materials and consumer products often pose a threat to the environment safety and human health [2–13]. According to World Health Organization in 2004, American California in 2008, and European Union in 2014, the formaldehyde has been categorized as a human carcinogen prone to some serious diseases such as the nasopharyngeal cancer or the leukemia, thereby raising widespread scientific and regulatory concerns [14]. To understand and control this issue, the research on product formaldehyde emissions has become a hot interdisciplinary topic in the fields of material and environmental sciences [1–81].

Generally speaking, the formaldehyde emitted from wood building materials is one of the main reasons that cause a poor indoor air quality, which is largely due to the widespread usage of synthetic resin adhesives in wood products, such as the urea-formaldehyde (UF) resin, the phenol-formaldehyde (PF) resin, and the melamine-urea-formaldehyde (MUF) resin [15–18]. Especially the urea-formaldehyde resin, it often features the low cost, simple process, fast curing, water solubility, and desirable physical and mechanical properties, thus being economically important in the forest products industry [19]. To improve the performance of wood adhesives, the formaldehyde is typically added in excess in the resin, thus forming the emittable formaldehyde at a room temperature [20]. Compared to the diminutive amount of the formaldehyde naturally occurring in the solid wood, the formaldehyde emission behavior of synthetic resin adhesives plays a more important role in the indoor air pollution caused by wood building materials [21].

To understand and control the product formaldehyde emission, lots of fascinating work has been done, which often involves the study on the emission reduction and the risk assessment. For the emission reduction, it can be achieved in different stages of the product manufacture and usage. For example, some formaldehyde-free adhesives such as the polyolefin, the poly (vinyl alcohol), the brewer’s yeast biomass, and the bacterial cellulose can provide a substitute for the urea-formaldehyde resin to fabricate wood composites [22–25]. Besides, the physical and chemical modifications for synthetic resin adhesives are also feasible. For the physical modification, fillers with a tunnel release effect like the sepiolite and the nanocrystalline cellulose can be added in the resin to diminish the formaldehyde emission; while for the chemical modification, the synthesis procedure of resin adhesives can be adjusted by some approaches, such as changing the molar ratio and the addition order of raw materials, or supplementing some solvents like the ethanol [26–33]. Furthermore, the moulding parameters for wood composites affect the formaldehyde emission, such as the assembly time, the hot-pressing process, and the cure conditions [34,35]. During the indoor application, some further treatments like the edge sealing and the surface finishing can be adopted to control the product formaldehyde emission too [36,37].

As for the risk assessment, characterization of the product formaldehyde emission has received great concerns. On one hand, since the formaldehyde emission behavior can be simulated and predicted combining mass transfer models and emission characteristic parameters, plenty of studies on the model development and the parameter estimation have been carried out [38–46]. On the other hand, to facilitate the quality supervision and inspection for wood products, various standard test methods for the product formaldehyde emission have been issued by governments or international organizations, and some popular standard test methods include the European EN120 (the perforator extraction method), EN 717–1 (the environmental chamber method), EN 717–2 (the gas analysis method), EN 717–3 (the flask method), ENV 13419–2 (the field and laboratory emission cell method); the American ASTM D 5582 (the desiccator method), ASTM D 6007–2 and ASTM E 1333 (the environmental chamber method); and the Japanese JIS A 1460 (the desiccator method), most of which are proposed by developed countries and regions [47–49]. Usually, the formaldehyde emission of a material can be characterized by different standard test methods, while a standard test method can also be employed to characterize the formaldehyde emission of different materials [50–51]. Compared to the simulation-based strategies for estimating product formaldehyde emissions, for prudence, these standard test methods for the risk assessment would be more practical and feasible in the production control.

In recent years, the research on standard test methods for the product formaldehyde emission has attracted increasing attentions from a wide range of scientific interests, which often involves the investigation on product emission levels, measurement uncertainties, and data correlations between various methods. For one thing, standard test methods can be used to recognize the formaldehyde emission behavior of indoor materials and products. For example, the formaldehyde emission level of a variety of solid woods, wood based panels, and finishing products have been characterized by many standard test methods [52–55]. Especially, since the formaldehyde emission value can be expressed as a gas-phase concentration by the environmental chamber method, this method has been universally considered the most reliable and accurate way for evaluating the effect of product formaldehyde emissions on the indoor air quality [47]. For another, the measurement uncertainty can provide a useful indication of the level of accuracy of the formaldehyde emission value characterized by standard test methods. To elucidate the representativeness and the reliability of the most probable value in a measurement, the uncertainty can be reflected through many ways, such as the histogram of data distribution, the correlation coefficient, the coefficient of determination, the coefficient of variation, and the result of the inter-laboratory comparison [56–59]. For instance, the Monte Carlo method has become a popular approach to analyze the measurement uncertainty [60–62]. On the other hand, the study on data correlations of formaldehyde emission values characterized by different methods and their influential factors would contribute to the technology exchange and collaboration around the world. For example, the perforator extraction method is conventionally employed in Europe, and the desiccator method is widely adopted in Asia-Pacific region, while the different environmental chamber methods have been standardized in Europe and North America; in this sense, the differences in procedures and details between standard test methods would make researchers and manufacturers at different regions of the world difficult to compare and understand the results of each other, thus underscoring a necessity for establishing the empirical correlations [63–71].

However, the available reports on standard test methods for product formaldehyde emissions mainly focus on the methods of developed countries and regions, particularly the EN system, the ASTM system, and the JIS system of Europe, America and Japan; in contrast, the standard test methods of developing countries like China have been barely mentioned. De facto, combining the advanced experience of developed countries and regions with the industrial status of China, a mandatory national standard GB 18580 has recently been proposed by Chinese State Forestry Administration to limit the formaldehyde emission of wood building materials towards the indoor decorating and refurbishing application, which contains 4 standard test methods (the perforator extraction method, the 9–11 L desiccator method, the 40 L desiccator method, and the environmental chamber method) that are not exactly the same as the existing methods of developed countries and regions. Moreover, the research on standard test methods is very necessary for China, whose indoor air quality is encountering a great challenge caused by product formaldehyde emissions [72–77]. During the last decades, the Chinese formaldehyde industry has experienced an unprecedented growth, which currently accounts for more than 33% of the formaldehyde production and consumption in the world; in detail, over two-thirds of the Chinese formaldehyde output often serves the manufacture of synthetic resin adhesives for wood products—the major source of the indoor air pollution in China [78]. In this context, the boom of the Chinese forest products industry substantially aggravates the indoor air pollution caused by product formaldehyde emissions. In recent years, the yield of wood composites in China has also undergone a rapid increase, whose annual growth rate is averagely over 20% since 2000, thus making China the largest producer, consumer, and trader in the world; taking the production of wood based panels as an example, it has risen from about 5 × 10^7^ m^3^ in 2004 to over 30 × 10^7^ m^3^ in 2014 [79]. As the largest formaldehyde producer and consumer, the Chinese population is potentially at increased risk for formaldehyde-induced health problems (*e*.*g*., the poisoning, cancer, and other associated effects), which has exceeded the acceptable benchmark [80]. However, a lack of systematic investigations and basic data on formaldehyde emission characteristics in Chinese standard tests would hinder the development of the risk assessment for product formaldehyde emissions.

To deepen the insight into the emission reduction and the risk assessment for product formaldehyde emissions, various attempts have been recently made by the authors’ team [22–24,34,81], and this work would present a first effort to focus on the formaldehyde emission characteristics of wood building materials in Chinese standard tests, whose main contributions were as follows:

1. Formaldehyde emission behaviors of 5 wood building materials (fiberboards, particleboards, blockboards, floorings, and parquets) were investigated by 4 test methods (the perforator extraction method, the 9–11 L desiccator method, the 40 L desiccator method, and the environmental chamber method) of the Chinese national standard GB 18580.
2. According to the limits of the Chinese GB 18580, the formaldehyde emission level and the product quality of these wood building materials that were manufactured in Beijing region (*i*.*e*., the capital of China) were assessed, while important features of these standard test methods were also recognized through comparing with existing methods and available reports.
3. The statistical distributions of formaldehyde emission data in multiple tests were determined, based on which the measurement uncertainty in Chinese standard tests for product formaldehyde emissions was evaluated applying the Monte Carlo method. In terms of statistics, this work can elucidate the representativeness of the most probable value, as well as the reliability of the measurement.
4. By virtue of 2 mathematical models, empirical correlations for the formaldehyde emission value characterized by various Chinese standard test methods were established, while the scope of application for these models was also revealed, which can provide a basis for the further comparison of standard test methods between China and developed countries and regions in the future research, thus promoting the technology exchange and collaboration around the world.

## Materials and Methods

### Wood building materials for formaldehyde emission tests

As shown in Tables 1 and 5 common wood building materials (fiberboards, particleboards, blockboards, floorings, and parquets) were considered for formaldehyde emission tests of this research, which were provided by the Chinese National Center for Quality Supervision and Inspection of Furniture and Indoor Environment. In detail, these products were sampled in Beijing region (*i*.*e*., the capital of China), which were all newly produced, and made from the poplar species and the urea-formaldehyde resin adhesive.

**Table 1:** Wood Building Materials for Formaldehyde Emission Tests of This Research.

Wood building material	Specification (mm)	Raw material
Medium density fiberboard (MDF)	2460 × 1250 × 18	Wood fiber + Adhesive
Particleboard (PB)	2460 × 1250 × 18	Wood particle + Adhesive
Blockboard (BB)	2460 × 1250 × 17	Surface wood veneer + Core wood strip + Adhesive
Laminate flooring (LF)	1220 × 202 × 12	Resin impregnated paper + Core particleboard + Adhesive
Parquet (PQ)	1210 × 192 × 15	Surface wood veneer + Core plywood + Adhesive

### Test, sampling and analysis

**Fig 1:**
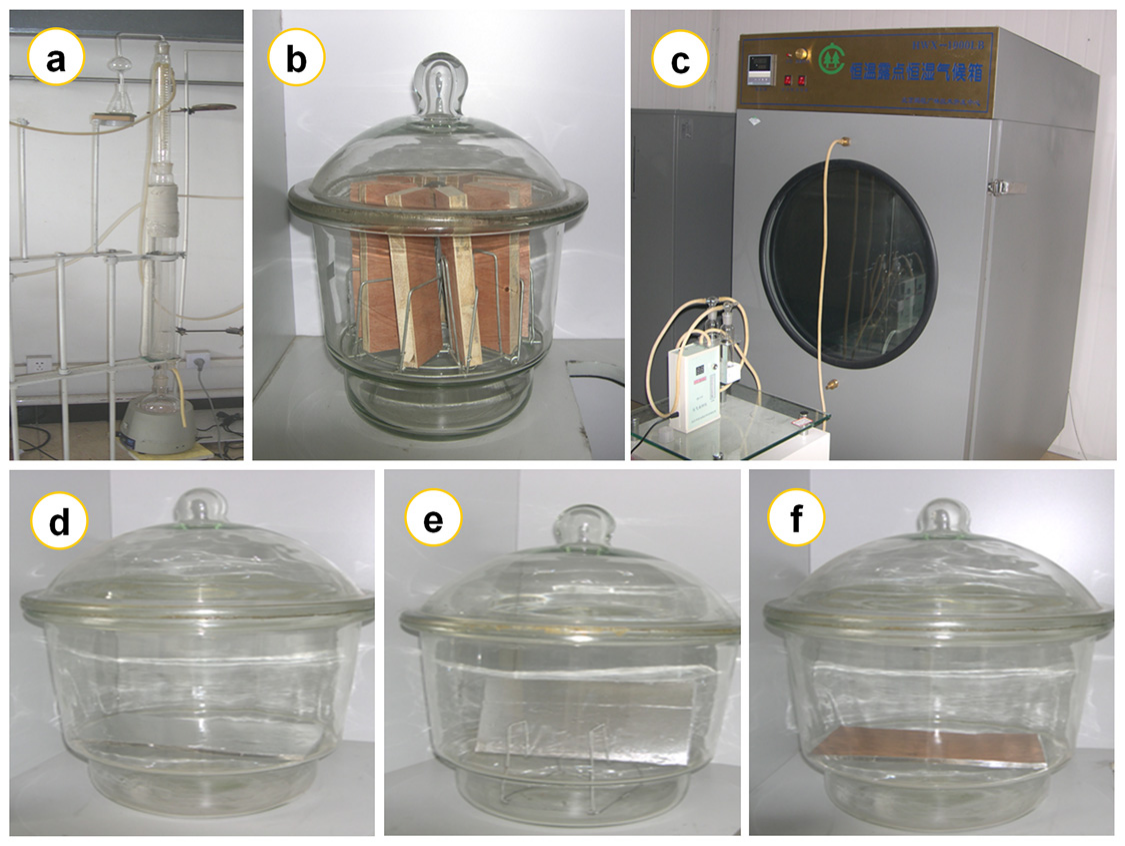
The 4 standard test methods of the Chinese national standard GB 18580 for product formaldehyde emissions. (a) The perforator extraction method. (b) The 9–11 L desiccator method. (c) The environmental chamber method. (d) The 40 L desiccator method (Posture A, the 450 cm^2^ emission surface of the specimen faced the distilled water at the bottom of the desiccator for sampling). (e) The 40 L desiccator method (Posture B, the 450 cm^2^ emission surface of the specimen faced the distilled water with 45°). (f) The 40 L desiccator method (Posture C, the sealed surface of the specimen faced the distilled water). About the consideration of the 3 situations for the 40 L desiccator method, see the corresponding results and discussion for the 40 L desiccator method.

As illustrated in Fig 1, formaldehyde emission characteristics of the 5 wood building materials were respectively investigated by 4 test methods of the Chinese national standard GB 18580 “Indoor Decorating and Refurbishing Materials—Limit of Formaldehyde Emission of Wood-based Panels and Finishing Products”, which included the perforator extraction method, the 9–11 L desiccator method, the 40 L desiccator method, and the environmental chamber method, thus resulting in 20 material-method combinations (= 5 materials × 4 methods) in this research. For each wood building material (of the 5 material), specimens used in the 4 formaldehyde emission tests were identical, which were from the same batch. Depending on standard test methods, the proper size and number of specimens were prepared from these full-size panels in Table 1, and then used for corresponding formaldehyde emission tests.

For all the 4 Chinese standard tests, the formaldehyde of wood building materials was sampled by the distilled water, while the solution was then analyzed by the acetylacetone spectrophotometry. Based on a highly specific Hantzsch reaction, the aqueous formaldehyde may react with the mixture of the acetylacetone and the ammonium ion, thus yielding the 3,5-diacetyl-1,4-dihydrolutidine (DDL). Subsequently, the formaldehyde emission value can be calculated from the solution absorbance, which was read through a UV-vis spectrophotometer at a wavelength of 412 nm.

For the 4 Chinese standard tests, more details about the specimen preparation, and test and sampling in this research can be found in corresponding sections of “Results and Discussion” (such as Table 2 for the perforator extraction method, Table 3 for the 9–11 L desiccator method and the 40 L desiccator method, Table 4 for the environmental chamber method, and their corresponding discussion), where systematic comparisons on the technical details between standard test methods of China and those of developed countries and regions were further made.

**Table 2:** Characterization of the Product Formaldehyde Emission: The Perforator Extraction Methods in Popular Standards and Chinese GB 18580.

	Specimen preparation	Test and sampling	Analysis
EN 120	1. Each piece: 25 mm × 25 mm × *t*. 2. Specimens used in the test: About 110 g.	1. The formaldehyde extraction: From the material to the toluene, 110.8°C for 2 h. 2. The formaldehyde sampling: From the toluene to the distilled water.	TAS
GB 18580	1. Each piece: 20 mm × 20 mm × *t*. 2. Specimens used in the test: 105 g to 110 g.	1. The formaldehyde extraction: From the material to the toluene, 110.8°C for 2 h. 2. The formaldehyde sampling: From the toluene to the distilled water.	TAS, or TI. In this research: TAS

(a) *t*, the product thickness.

(b) TAS, the acetylacetone spectrophotometry, which is prior to products with a low emission.

(c) TI, the iodometry.

**Table 3:** Characterization of the Product Formaldehyde Emission: The Desiccator Methods in Popular Standards and Chinese GB 18580.

	Specimen preparation	Specimen edge	Desiccator
ASTM D 5582	1. Each piece: 127 mm × 70 mm × *t*. 2. Specimens used in the test: 8 pieces.	Sealed, with the liquid paraffin	9–11 L
JIS A 1460	1. Each piece: 150 mm × 50 mm × *t*. 2. Specimens used in the test: About 1800 cm^2^ emission surface, including the exposed edge.	Exposed, and the area is included in the emission surface area	9–11 L
GB 18580 (the 9–11 L desiccator method)	1. Each piece: 150 mm × 50 mm × *t*. 2. Specimens used in the test: 10 pieces.	Exposed	9–11 L
GB 18580 (the 40 L desiccator method)	1. Each piece: Not required. In this research: 300 mm ×150 mm × *t*, with a single-side emission, and the other side was sealed with the aluminum tape. 2. Specimens used in the test: 450 cm^2^ emission surface. In this research: 1 piece.	Sealed, with the aluminum tape	40 L
	Conditioning	Test and sampling	Analysis
ASTM D 5582	1. Conditions: 24°C, 50% humidity. 2. The period: 7 days.	1. Test: 24°C, 2 h. 2. Sampling: By 25 mL distilled water.	TCAS
JIS A 1460	1. Conditions: 20°C, 65% humidity. 2. The period: ≤ 7 days.	1. Test: 20°C, 24 h. 2. Sampling: By 300 mL distilled water.	TAS
GB 18580 (the 9–11 L desiccator method)	Not required	1. Test: 20°C, 24 h. 2. Sampling: By 300 mL distilled water.	TAS
GB 18580 (the 40 L desiccator method)	1. Conditions: 20°C, in the vinyl resin bag. 2. The period: ≥ 1 day.	1. Test: 20°C, 24 h. 2. Sampling: By 20 mL distilled water.	TAS

(a) *t*, the product thickness.

(b) TAS, the acetylacetone spectrophotometry.

(c) TCAS, the chromotropic acid spectrophotometry.

**Table 4:** Characterization of the Product Formaldehyde Emission: The Environmental Chamber Methods in Popular Standards and Chinese GB 18580.

	Specimen preparation	Specimen edge	Chamber
ASTM D 6007–02	1. Each piece: Not required. 2. Specimens used in the test: The loading factor is 0.13 to 0.95 m^2^/m^3^.	Sealed, with the aluminum tape, if the exposed edge area ≥ 5% of the emission surface area	0.02 to 1 m^3^
ASTM E 1333	1. Each piece: Not required. 2. Specimens used in the test: The loading factor is 0.13 to 0.95 m^2^/m^3^.	Exposed, and the area is included in the emission surface, if the exposed edge area ≥ 5% of the emission surface area	≥ 22 m^3^
EN 717–1	1. Each piece: 0.2 m × 0.28 m × *t* for the 0.225 m^3^ chamber, 0.5 m × 0.5 m × *t* for the 1 m^3^ chamber, 1.0 m × 2.0 m × *t* for the large chamber. 2. Specimens used in the test: The loading factor is 1 m^2^/m^3^.	Partly sealed, making the ratio of the exposed edge length to the emission surface area = 1.5 m/m^2^ for the small chamber (0.225 m^3^ and 1 m^3^)	0.225 m^3^, or 1 m^3^, or the large chamber (≥ 12 m^3^ and in multiples of 4 m^3^)
GB 18580	1. Each piece: Not required. 1 m × 0.5 m × *t*, or 0.5 m × 0.5 m × *t* are recommended. In this research: 1000 mm × 500 mm × *t* for the fiberboard, particleboard, and blockboard; and 1000 × 125 mm × *t* for the flooring and parquet. 2. Specimens used in the test: The loading factor is 1 m^2^/m^3^. In this research: 1 piece for the fiberboard, particleboard, and blockboard; and 4 pieces for the flooring and parquet.	Sealed, with the aluminum tape	1 m^3^
	Conditioning	Test and sampling	Analysis
ASTM D 6007–02	1. Conditions: 24°C, 50% humidity. 2. The period: 2 h.	1. Test: 25°C, 50% humidity, the ratio of the ventilation volume to the emission surface area = 0.526 to 3.846 m/h, till a steady-state emission. 2. Sampling: By the distilled water.	TCAS
ASTM E 1333	1. Conditions: 24°C, 50% humidity. 2. The period: 7 d.	1. Test: 25°C, 50% humidity, 0.5 h^-1^ air change rate, till a steady-state emission. 2. Sampling: By the distilled water.	TCAS
EN 717–1	Not required	1. Test: 23°C, 45% humidity, 1 h^-1^ air change rate, 10 days to 28 days. 2. Sampling: By the distilled water.	TAS
GB 18580	Not required	1. Test: 23°C, 45% humidity, 1 h^-1^ air change rate, 10 days to 28 days. 2. Sampling: By the distilled water.	TAS

(a) *t*, the product thickness.

(b) TAS, the acetylacetone spectrophotometry.

(c) TCAS, the chromotropic acid spectrophotometry.

### Experimental design

To performed a systematic investigation on formaldehyde emission characteristics of wood building materials in Chinese standard tests, this research would conduct the survey on “product emission levels”, “measurement uncertainties”, and “data correlations between various methods”. Considering that, the experiments in this research were designed as follows:

As recognized in preliminary studies, when the number of formaldehyde emission data reached about 60, statistical distributions for the data of the 20 material-methods combinations in this research (= 5 materials × 4 methods) can be reflected. Therefore, to improve the accuracy of results and facilitate the measurement uncertainty evaluation that need enough data, 60 was selected as the sample size (the required number of formaldehyde emission data) for each material-method combination.

For one thing, for the perforator extraction method, the 9–11 L desiccator method, and the 40 L desiccator method, 60 tests were respectively carried out for each material-method combination (of the 15 combinations = 5 materials × 3 methods), thus deriving 60 formaldehyde emission values for each material-method combination that first served the measurement uncertainty evaluation. Then, the arithmetic mean of 60 formaldehyde emission values was calculated to represent the final result of the formaldehyde emission value for the corresponding material-method combination that was used to assess the product emission level, which was also employed when finally establishing data correlations between various tests.

For another, for the environmental chamber method, the measurement for each material (of the 5 materials) respectively lasted 12 days, during which 5 gas-phase formaldehyde concentrations in the chamber air were daily sampled for each material (each concentration was the arithmetic mean of 2 duplicate samples), thus deriving 60 formaldehyde emission values for each material that first served the measurement uncertainty evaluation. Then, the arithmetic mean of formaldehyde concentrations at the 264 h (11 day) and the 288 h (12 day) was calculated to represent the final result of the formaldehyde emission value for the corresponding material that was used to assess the product emission level, which was also employed when finally establishing data correlations between various tests. For the 5 materials studied, their coefficients of variation for formaldehyde emission concentrations in the chamber air between the 264 h (11 day) and the 288 h (12 day) were all below 5%, which met the criterion of the Chinese national standard GB 18580 for a steady-state emission, thus indicating that all the formaldehyde emission tests can be ended at this time.

## Results and Discussion

### Product emission levels in Chinese standard tests

#### Product formaldehyde emissions in the Chinese perforator method

The perforator extraction method is conventionally applied in European countries, which is developed by former European Particleboard Federation in the late 1960s, and then listed in the European standard EN 120 in 1984 [49]. In a typical run, the formaldehyde in small specimens is first extracted by the boiling toluene, and the formaldehyde in this solution is further extracted (sampled) by the distilled water; finally, the aqueous formaldehyde is analyzed by the acetylacetone spectrophotometry [38]. Due to the comparably simple equipment and a short total running time (about 3 h), this method has been widely accepted in the production control of the forest products industry, especially for unlaminated and uncoated materials with the overall similar structure and density [48]. As shown in Table 2, the perforator extraction method of the Chinese GB 18580 is slightly different from that of the European EN 120, such as the dimension of the test piece.

**Fig 2:**
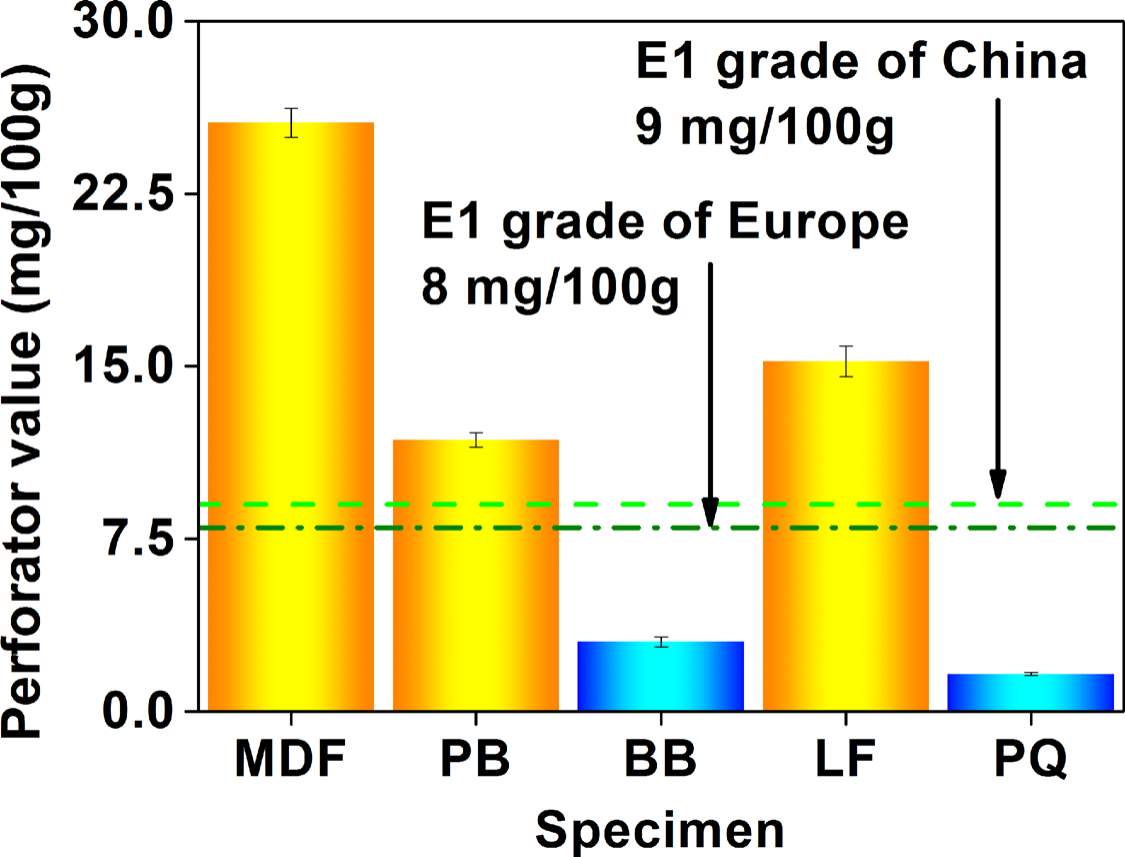
Formaldehyde emission values (perforator value) of various materials characterized by the perforator extraction method of the Chinese national standard GB 18580. MDF, medium density fiberboard; PB, particleboard; BB, blockboard; LF, laminate flooring; PQ, parquet.

As illustrated in Fig 2, the perforator test value (the formaldehyde emission value characterized by the perforator extraction method, whose unit is “mg (formaldehyde)/100g (dry material)”) of the 5 wood building materials was in the range of 1 mg/100g to 26 mg/100g. To evaluate the product quality, a E1 grade (the perforator test value ≤ 9 mg/100g) and a E2 grade (the perforator test value ≤ 30 mg/100g) have been specified by the Chinese GB 18580 and the European EN 120, in which the product of the E1 grade can be directly used in the indoor environment [21]. In this context, the blockboard and parquet can meet the E1 grade with the perforator test value below 4 mg/100g, while the fiberboard, particleboard, and flooring with the perforator test value of 11 mg/100g to 26 mg/100g were just products of the E2 grade. Since the perforator test value is closely related to the content of the free formaldehyde in wood composites, the different perforator test values for various products in this research would be due to their different formulae and manufacturing processes, which can introduce and form the different amounts of the free formaldehyde [37,52,55]. Similar to previous reports using the perforator extraction method of the European EN 120, this research employing the perforator extraction method of the Chinese GB 18580 also found that the formaldehyde emission value of the fiberboard would be generally higher than that of the particleboard, which further reflected the reliability and the accuracy of Chinese standard test methods [52,63,64].

However, the perforator extraction method might have some drawbacks, such as the high-temperature extraction step. With an adsorption effect of the material microstructure, the existence of the free formaldehyde in wood composites often consists of a bound part and an emittable part, while only the latter would be guilty of the product formaldehyde emission and the indoor air pollution [43–45]. Generally speaking, most free formaldehyde in wood composites belongs to the bound part at a room temperature and never migrates, but the emittable formaldehyde seems very susceptible to a temperature change [46]. Taking the fiberboard as an example, with a temperature rise from 25°C to 80°C, the ratio of the emittable formaldehyde to the total formaldehyde (the bound part + the emittable part) increases from below 5 wt% to almost 70 wt%, while this change is explained by a statistical physics theory [38]. In this sense, despite the convenience of a perforator extraction method, the high-temperature extraction step would be significantly different from the indoor application, which may affect material physicochemical properties (especially the emittable content of the formaldehyde), possibly resulting in a misjudgment on the product quality. For instance, the particleboard with the perforator test value of the E1 grade can still lead to the chamber test value of the E2 grade, while the environmental chamber method has been universally considered the most reliable and accurate method [54,63].

#### Product formaldehyde emissions in the Chinese desiccator method

The desiccator method is widely adopted in countries of Asia-Pacific region, such as Japan, Korea, Indonesia, Malaysia, Australia, and New Zealand [21]. In a typical run, specimens are positioned in an airtight desiccator to emit the formaldehyde, and some formaldehyde is absorbed (sampled) by the distilled water in a container at the bottom of the desiccator; finally, the aqueous formaldehyde is analyzed by the acetylacetone spectrophotometry [19]. Compared to the perforator extraction method, the desiccator method would allow more specimens to be investigated at a room temperature [48]. As shown in Table 3, many details in the 4 desiccator methods of the 3 countries are different; for instance, the American desiccator method features a shorter duration (*i*.*e*., 2 h), while a bigger desiccator (*i*.*e*., 40 L) is considered by China.

**Fig 3:**
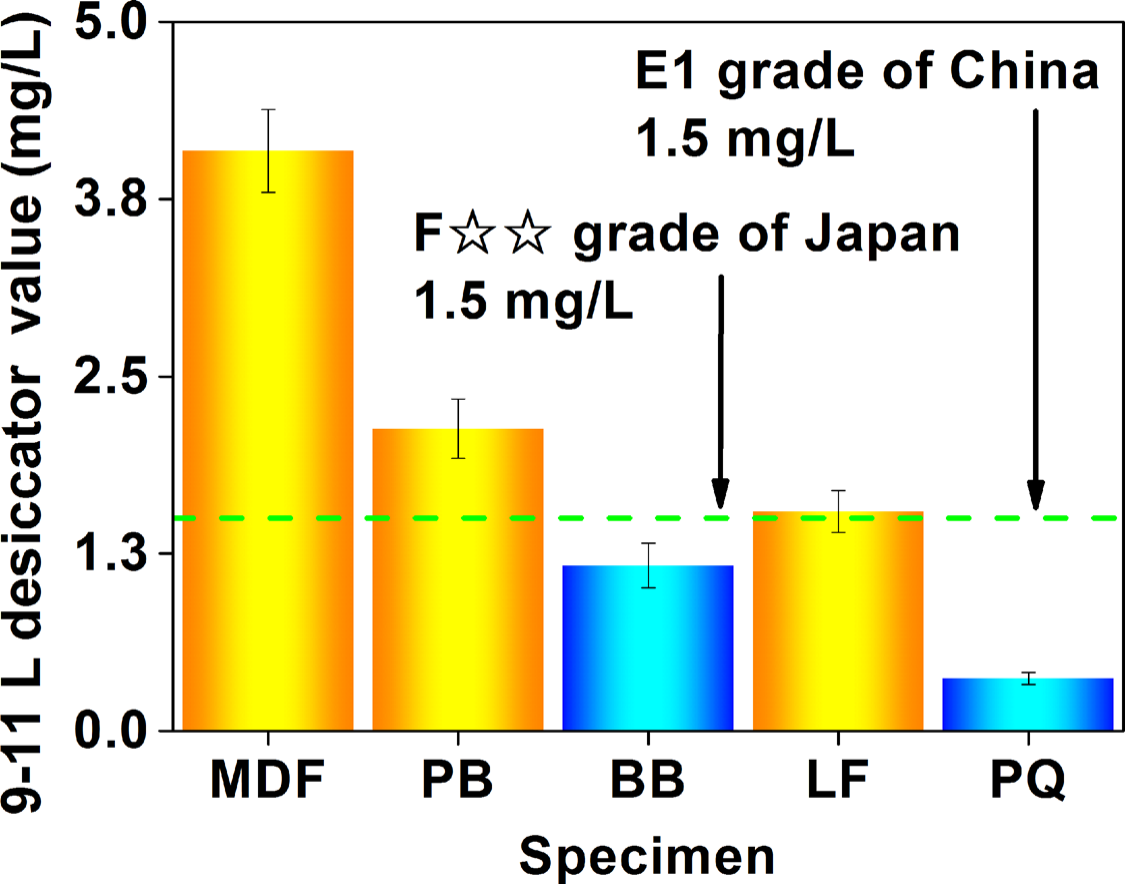
Formaldehyde emission values (9–11 L desiccator value) of various materials characterized by the 9–11 L desiccator method of the Chinese national standard GB 18580. MDF, medium density fiberboard; PB, particleboard; BB, blockboard; LF, laminate flooring; PQ, parquet.

As displayed in Fig 3, the 9–11 L desiccator test value (the formaldehyde emission value characterized by the 9–11 L desiccator method, whose unit is “mg (formaldehyde)/L (distilled water)”) of the 5 wood building materials was in the range of 0.3 mg/L to 4.1 mg/L. To evaluate the product quality, a E1 grade (the 9–11 L test value ≤ 1.5 mg/L) and a E2 grade (the 9–11 L test value ≤ 5.0 mg/L) have been specified by the Chinese GB 18580, in which the product of the E1 grade can be directly used in the indoor environment; besides, a F☆☆ grade in the Japanese JIS A 1460 is equivalent to a E1 grade [54]. In this context, the blockboard and parquet can meet the E1 grade with the 9–11 L desiccator test value under 1.2 mg/L,while the fiberboard, particleboard, and flooring with the 9–11 L desiccator test value of 1.5 mg/L to 4.1 mg/L were just products of the E2 grade. Clearly, products of the E1 grade (in the 5 materials in this research) identified by the 9–11 L desiccator method (the E1 grade products = the blockboard and the parquet) were the same as those identified by the perforator extraction method (as illustrated in Fig 2, and the corresponding discussion), thus implying their higher similarity [49]. Like previous reports using the 9–11 L desiccator method of the Japanese JIS A 1460 and the American ASTM D 5582, this research adopting the 9–11 L desiccator method of the Chinese GB 18580 also found that the formaldehyde emission value of the fiberboard would be generally higher than that of the particleboard, which further reflected the reliability and the accuracy of Chinese standard test methods [37,50,63,64,69,70].

Nevertheless, the loading factor (a ratio of the area for the formaldehyde emission to the volume for the test container) of the 9–11 L desiccator method is close to 20 m^2^/m^3^, which is much larger than that of general Chinese bedrooms (the loading factor = 0.42 ± 0.04 m^2^/m^3^) and living rooms (the loading factor = 0.23 ± 0.02 m^2^/m^3^) [16,58]. With that in mind, a novel standard test method based on a 40 L desiccator has been proposed by the Chinese national standard GB 18580, thus reducing the loading factor to about 1 m^2^/m^3^. De facto, the loading factor of the 40 L desiccator method is also very close to that of most popular environmental chamber methods (*e*.*g*., the European EN 717–1, and the American ASTM D 6007–02 and ASTM E 1333), which have been universally considered the most reliable and accurate method [21].

**Fig 4:**
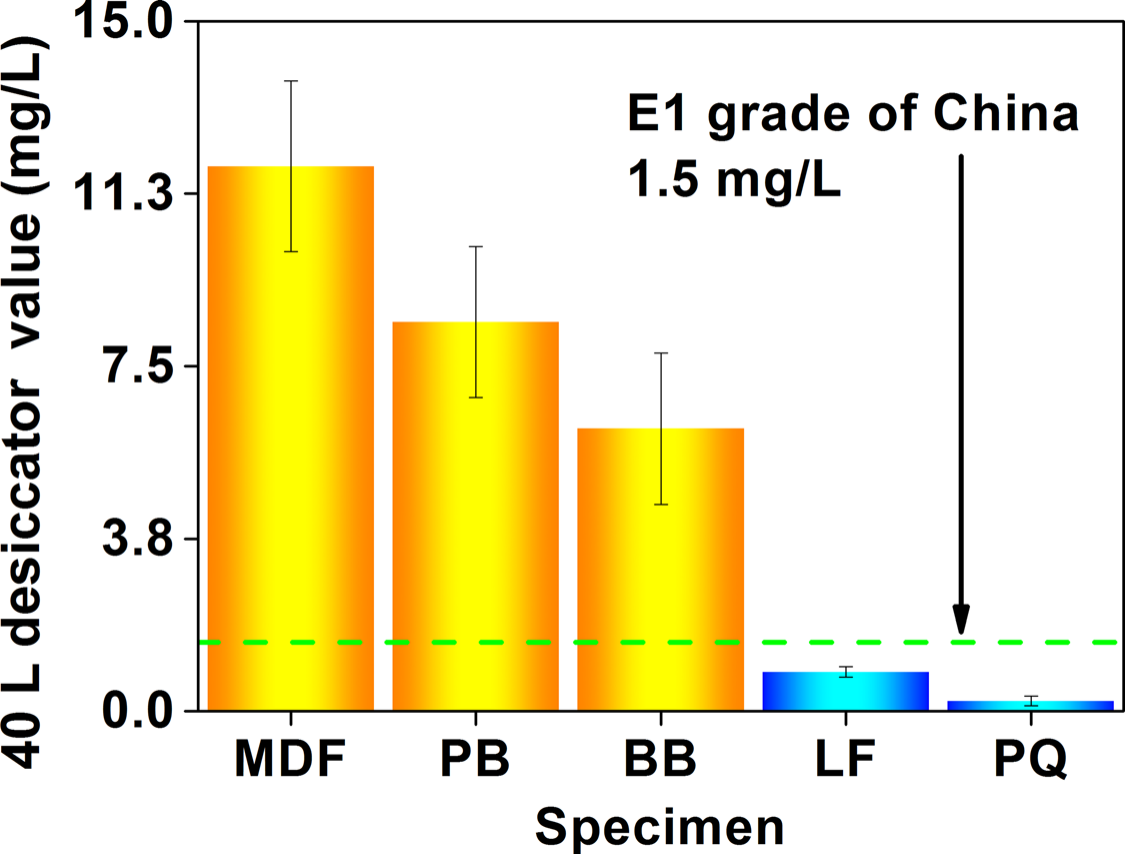
Formaldehyde emission values (40 L desiccator value) of various materials characterized by the 40 L desiccator method of the Chinese national standard GB 18580. MDF, medium density fiberboard; PB, particleboard; BB, blockboard; LF, laminate flooring; PQ, parquet.

As depicted in Fig 4, the 40 L desiccator value (the formaldehyde emission value characterized by the 40 L desiccator method, whose unit is “mg (formaldehyde)/L (distilled water)”) of the 5 wood building materials was in the range of 0.2 to 12 mg/L. To evaluate the product quality, a E1 grade (the 40 L desiccator test value ≤ 1.5 mg/L) and a E2 grade (the 40 L desiccator test value ≤ 5.0 mg/L) have been specified by the Chinese GB 18580, in which the product of the E1 grade can be directly used in the indoor environment. In this context, the flooring and parquet can meet the E1 grade with the 40 L desiccator test value lower than 0.9 mg/L, while the fiberboard, particleboard, and blockboard with the 40 L desiccator test value of 6 mg/L to 12 mg/L failed to attain the E2 grade. Obviously, products of the E1 grade (in the 5 materials in this research) identified by the 40 L desiccator method (the E1 grade products = the flooring and the parquet) were slightly different from those identified by the perforator extraction method and the 9–11 L desiccator method (the E1 grade products = the blockboard and the parquet, as illustrated in Figs 2 and 3, and the corresponding discussion), which would be caused by the larger scale of the 40 L desiccator test (*e*.*g*., the dimension of the test specimen, the volume of the test container); in contrast, products of the E1 grade (in the 5 materials in this research) identified by the 40 L desiccator method were the same as those identified by the following environmental chamber method (universally considered the most reliable and accurate method, see the corresponding results and discussion for the environmental chamber method), thus implying their higher similarity (such as the loading factor), and further reflecting the reliability and the accuracy of the Chinese 40 L desiccator method. In this sense, the “scale factor” of standard emission tests (*i*.*e*., the factor related to time and space scales of tests, such as the dimension of the test specimen, the volume of the test container, or the duration of the test procedure) seems indeed to play a role, in which a potential “boundary” may also exist between the small-scale tests (*e*.*g*., the perforator extraction method, and the 9–11 L desiccator method) and the medium-scale or large-scale tests (*e*.*g*., the 40 L desiccator method, and the environmental chamber method); in other words, formaldehyde emission values characterized by standard test methods at the different sides of this “boundary” may give slightly different evaluations on the product quality of the same material (*e*.*g*., a E1 grade and a E2 grade of the Chinese national standard GB 18580). As for the reason why the “scale factor” matters, it would be understandable as follows: the characteristic of formaldehyde emission value largely depends on the test (that obtains the value), whose important details and procedures consist of various “factors related to time and space scales”. Therefore, the “scale factor” is definitely responsible for the emission test and classification results, which may be a significant factor affecting some conclusions.

**Fig 5:**
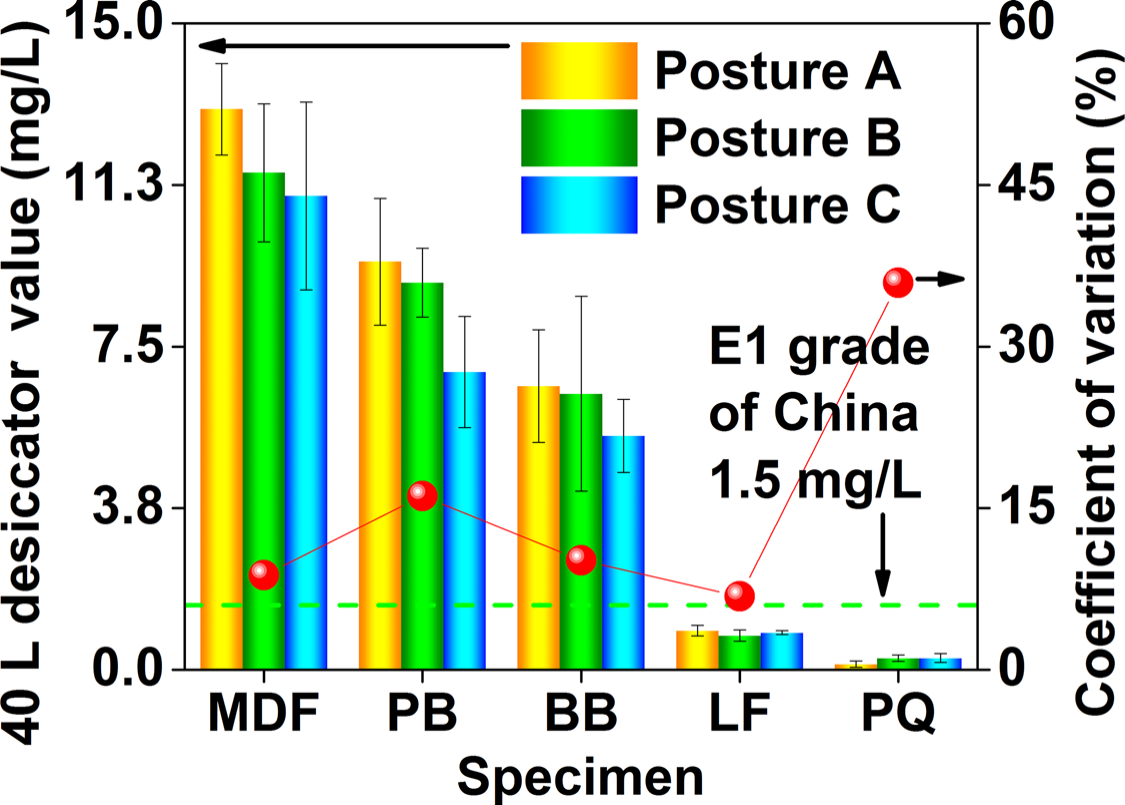
Formaldehyde emission values (40 L desiccator value) of various materials characterized by the 40 L desiccator method (under 3 situations of the specimen placement) of the Chinese national standard GB 18580. MDF, medium density fiberboard; PB, particleboard; BB, blockboard; LF, laminate flooring; PQ, parquet. Posture A, the 450 cm^2^ emission surface of the specimen faced the distilled water at the bottom of the desiccator for sampling; Posture B, the 450 cm^2^ emission surface of the specimen faced the distilled water with 45°; Posture C, the sealed surface of the specimen faced the distilled water. The coefficient of variation for each material (of the 5 materials) was caused by the formaldehyde emission values derived from the 3 situations of the specimen placement.

For the 40 L desiccator method, the Chinese national standard GB 18580 only specifies the specimen area for the formaldehyde emission—450 cm^2^; to consider the effect of the specimen placement on the formaldehyde emission value, the 60 tests of the 40 L desiccator method for each wood building material (of the 5 materials) were respectively divided into 3 groups as illustrated in Fig 1: 20 tests using the posture A (the 450 cm^2^ emission surface of the specimen faced the distilled water at the bottom of the desiccator for sampling), 20 tests using the posture B (the 450 cm^2^ emission surface of the specimen faced the distilled water with 45°), and 20 tests using the posture C (the sealed surface of the specimen faced the distilled water). As revealed in Fig 5, a difference in the specimen placement slightly affected the formaldehyde emission value; between the 3 postures, the coefficient of variation for the formaldehyde emission value of the 5 materials was in the range of 6% (the flooring) to 36% (the parquet). For most materials studied, the posture A and the posture C respectively led to a higher and a lower formaldehyde emission values, thus indicating an overall negative correlation between the formaldehyde emission value (of the 40 L desiccator method) and the included angle (between the emission surface of the specimen and the surface of the distilled water), probably because the formaldehyde would be easier to be absorbed (sampled) by the distilled water when the specimen gave a larger emission surface that faced the distilled water. However, the difference caused by the 3 postures of the specimen placement never changed the judgment of the 40 L desiccator method on products of the E1 grade in the 5 materials in this research (the E1 grade products = flooring and parquet), which further reflected the reliability and the accuracy of the Chinese 40 L desiccator method.

However, the desiccator method might have some drawbacks, such as the high humidity in the desiccator. Usually, the formaldehyde emission of wood composites would be affected by the introduction, aging, and hydrolysis of the urea-formaldehyde resin adhesive; while, featuring an irregular change with the time, the moisture-induced formaldehyde emission (related to the aging or the hydrolysis of adhesives) is very difficult to be quantitatively described [20]. But due to a excellent water solubility of the formaldehyde, the emission behavior of wood products is very susceptible to the dampness [48]. Taking the fiberboard as an example, the emitted formaldehyde concentration in a full-scale experimental room is observed to be positively correlated with the humidity that ranges over 1.1 g/kg_air_ to 23.1 g/kg_air_, while the emission behavior under the varied humidity (1.1 g/kg_air_ to 23.1 g/kg_air_) exhibits entirely different characteristics from that under constant environmental conditions [67]. In this sense, although the desiccator method avoids a high-temperature extraction step like the perforator extraction method, the increasing humidity in the airtight desiccator during tests caused by the distilled water for sampling would be significantly different from the indoor application, which may affect material physicochemical properties (especially the emittable content of the formaldehyde), possibly leading to a misjudgment on the product quality [64]. For instance, the particleboard and the parquet with the 9–11 L desiccator test value of the E1 grade can still result in the chamber test value of the E2 grade, while the environmental chamber method has been universally considered the most reliable and accurate method [63,65,70].

#### Product formaldehyde emissions in the Chinese chamber method

The environmental chamber method has been employed to characterize product formaldehyde emissions since the 1980s, which is currently standardized in Europe and North America [59]. In a typical run, specimens are placed in a test chamber with controlled environmental conditions (the temperature, humidity, and ventilation) to release formaldehyde, and the formaldehyde in the chamber air is then periodically pumped and absorbed (sampled) by the distilled water, until the formaldehyde concentration in the chamber air reaches a steady state; during the test, the aqueous formaldehyde is analyzed by the acetylacetone spectrophotometry [45]. Compared to the perforator extraction method and the desiccator method, the environmental chamber method is closer to the indoor application of materials and products, where the formaldehyde emission value can be expressed by a gas-phase concentration (mg/m^3^ or ppm) in the chamber air, thus facilitating the comparison with indoor air quality standards; therefore, according to the Chinese national standard GB 18580, the environmental chamber method should be used when conducting an arbitration [21]. As shown in Table 4, despite many differences in details and procedures between various environmental chamber methods, the 1 m^3^ chamber method seems acceptable for most standards.

**Fig 6:**
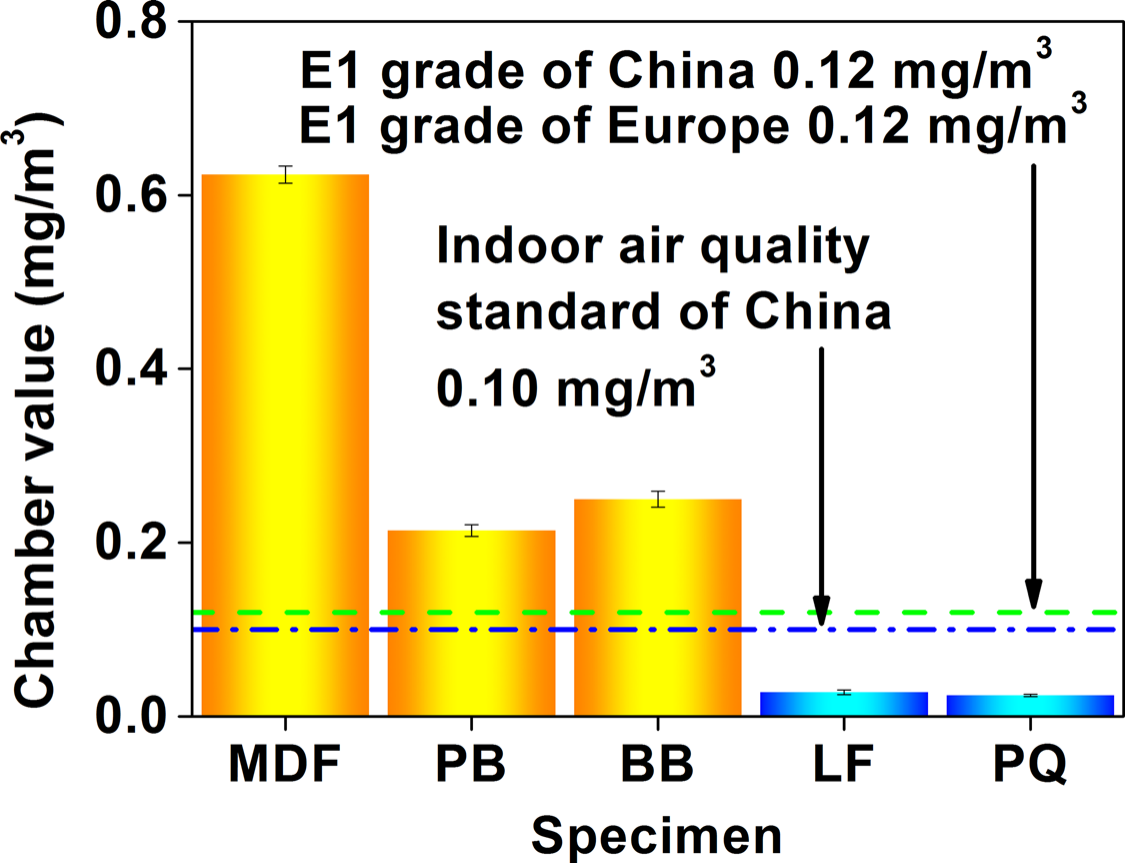
Formaldehyde emission values (chamber value) of various materials characterized by the environmental chamber method of the Chinese national standard GB 18580. MDF, medium density fiberboard; PB, particleboard; BB, blockboard; LF, laminate flooring; PQ, parquet.

As exhibited in Fig 6, the chamber test value (the formaldehyde emission value characterized by the environmental chamber method, whose unit is “mg (formaldehyde)/m^3^ (chamber air)”) of the 5 wood building materials was in the range of 0.02 mg/m^3^ to 0.62 mg/m^3^. To evaluate the product quality, a E1 grade (the chamber test value ≤ 0.12 mg/m^3^) has been specified by the Chinese GB 18580 and the European EN 717–1, and the product of the E1 grade can be directly used in the indoor environment [55]. In this context, the flooring and parquet can meet the E1 grade with the chamber test value below 0.03 mg/m^3^, while the fiberboard, particleboard, and blockboard with the chamber test value of 0.21 to 0.62 mg/m^3^ failed to achieve the E1 grade. Similar to the 40 L desiccator method (as illustrated in Fig 4, and the corresponding discussion), products of the E1 grade (in the 5 materials in this research) identified by the environmental chamber method (the E1 grade products = the flooring and the parquet) were slightly different from those identified by the perforator extraction method and the 9–11 L desiccator method (the E1 grade products = the blockboard and the parquet, as illustrated in Figs 2 and 3, and the corresponding discussion), and this fact (*i*.*e*., the difference between the perforator, desiccator, and chamber test values) coincided with some published results too [54,63,65,70]. Like previous reports using the environmental chamber method of the European EN 717–1, and the American ASTM D 6007–02 and ASTM E 1333, this research applying the environmental chamber method of the Chinese GB 18580 also found that the formaldehyde emission value would generally give a tendency of the fiberboard > the particleboard, the particleboard > the flooring, and the blockboard > the parquet, which further reflected the reliability and the accuracy of the Chinese standard test methods [53,59,63,64,69].

**Fig 7:**
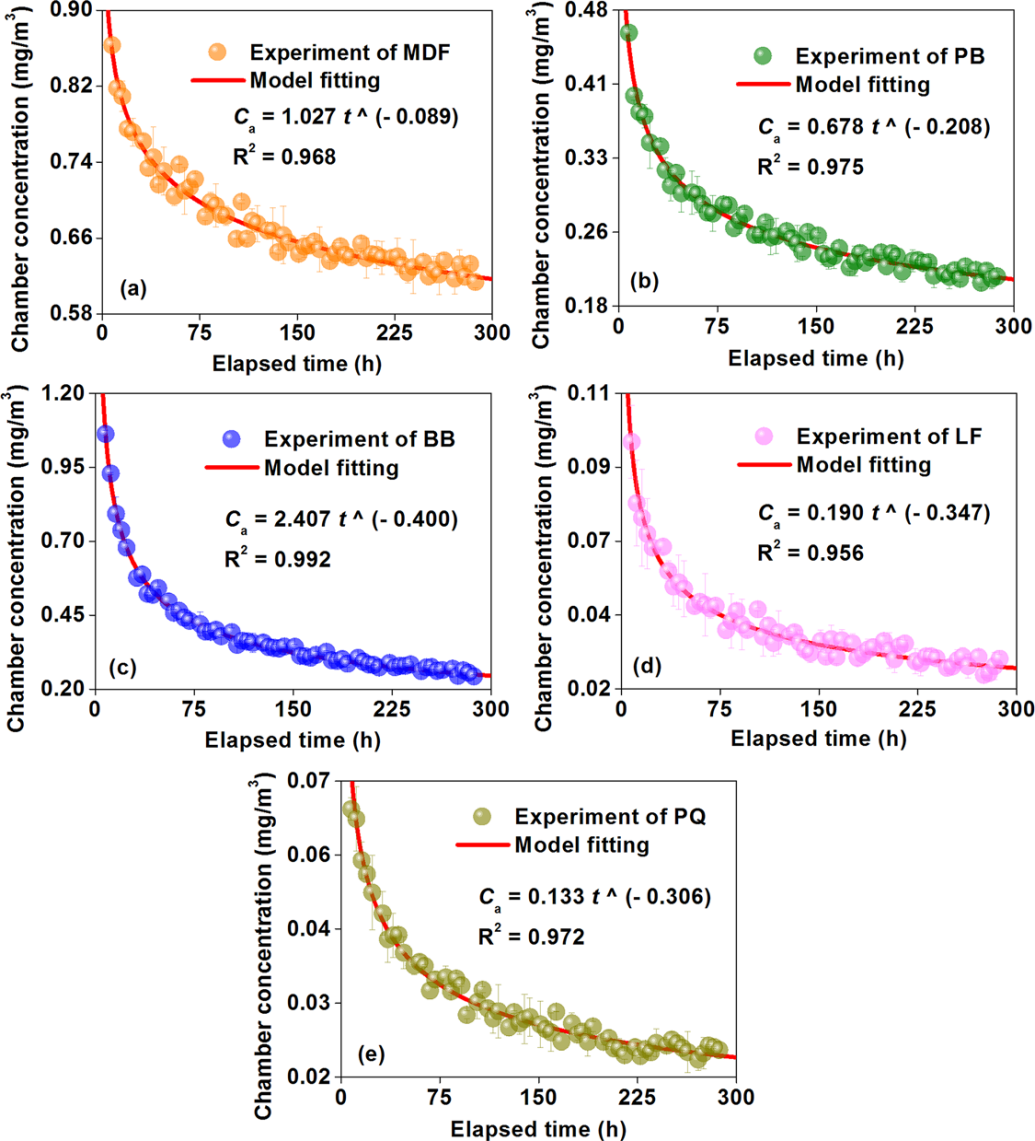
Formaldehyde emission concentrations in the chamber air (chamber concentrations *C*_a_) *vs*. the test time (or elapsed time *t*) of various materials characterized by the environmental chamber method of the Chinese national standard GB 18580. MDF: medium density fiberboard, PB: particleboard, BB: blockboard, LF: laminate flooring, PQ: parquet.

Although the environmental chamber method is widely acknowledged to be the most reliable and accurate method for the risk assessment of product formaldehyde emissions, it might also have some flaws, such as a higher device cost (*e*.*g*., constructing the larger chambers) and a longer test time (*i*.*e*., 7 to 28 days) [59]; however, some strategies can be considered to make the improvements. Compared to the perforator extraction method and the desiccator method, controlled climatic conditions (the temperature, humidity, and ventilation) of the environmental chamber method are particularly appropriate to perform simulation studies, which facilitates simplifying the formaldehyde emission test [20]. For one thing, some empirical associations have been proposed to correlate pollutant emission data under different loading factors (or air flow rates, and air change rates) [39]. In this sense, formaldehyde emission data can be conveniently derived from small chambers, and then converted into results for various full-scale experimental rooms that can better approximate product formaldehyde emissions in actual buildings, thus reducing the higher device cost of constructing larger chambers [42]. For another, with the increase of the time during chamber tests, Fig 7 indicated that the formaldehyde concentration changes of various materials in this research would undergo two major phases: an external convection-controlled rapid attenuation, and an internal diffusion-controlled steady state, which can be fitted by virtue of a power-law model of Eq (1), with coefficients of determination (R^2^) all over 0.95 [47]. According to the American ASTM D 5157 “Standard Guide for Statistical Evaluation of Indoor Air Quality Models”, a R^2^ ≥ 0.81 would provide a good indication of adequate model performance [38]. In this context, formaldehyde concentration data at the initial stage of an environmental chamber test can be taken to perform modeling, and the steady-state emission value can then be predicted, thus shortening the longer test time of 7 to 28 days [44,45]. In an aggregate, to be prudent, there is a need for these simulation-based strategies to receive more validation in the future.

Ca=β⋅t^α (1)

where *C*_a_ is the gas-phase formaldehyde concentration in the chamber air emitted from the wood building material, *t* is the test time, *α* and *β* are parameters for this model.

### Measurement uncertainties in Chinese standard tests

#### The uncertainty evaluation: Principle of the Monte-Carlo method

In terms of statistics, considering the difference between a limited number of “samples” (formaldehyde emission data in this research) and the corresponding “population”, there is a need to perform the uncertainty evaluation, which can elucidate the representativeness of the most probable value, as well as the reliability of the measurement [58]. With the development in computer science, the Monte Carlo method proposed in the 1940s has become a popular statistical simulation technology concerned with the numerical computation based on the probability theory [60]. Recently, this method has been successfully employed to assess the uncertainty of many researches, such as the survey of loading factors of wood products for Chinese residences, the calculation of mass transfer parameters for volatile organic compounds, the study of elastic properties for particles filled polymers, and the analysis of stress variations for composite single lap joints [39,61,62]. In a typical run of the Monte Carlo method, the uncertainty of a measurement can be evaluated as follows [16]:

1. Based on the distribution of survey data (*i*.*e*., the “sample” in statistics), build an appropriate mathematical model to describe their statistical characteristics.
2. Combining the model and the computer, perform a large scale simulation survey, whose data can be used to approximate the corresponding “population” in statistics.
3. According to the “population” in statistics, define and calculate the measurement uncertainty in terms of the objective of a research, and then assess the results.

#### Step 1 of uncertainty evaluation: Data distribution and modeling

**Fig 8:**
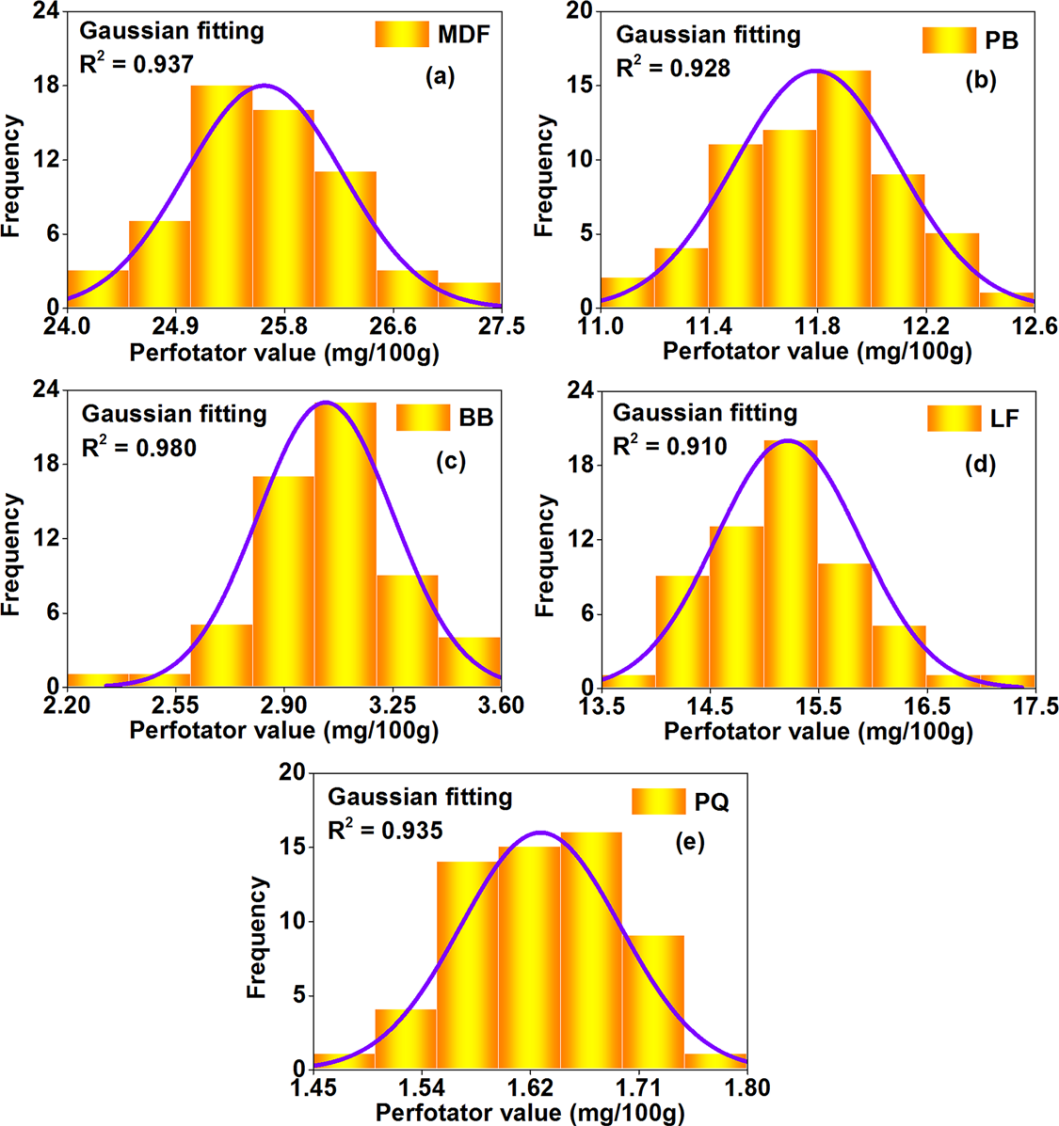
Statistical distributions of formaldehyde emission values (perforator value, derived from multiple tests) of various materials characterized by the perforator extraction method of the Chinese national standard GB 18580. (a) MDF, medium density fiberboard. (b) PB, particleboard. (c) BB, blockboard. (d) LF, laminate flooring. (e) PQ, parquet. The frequency denoted the number of data occurred at corresponding formaldehyde emission levels. These data were fitted by the probability density function of a Gaussian distribution.

**Fig 9:**
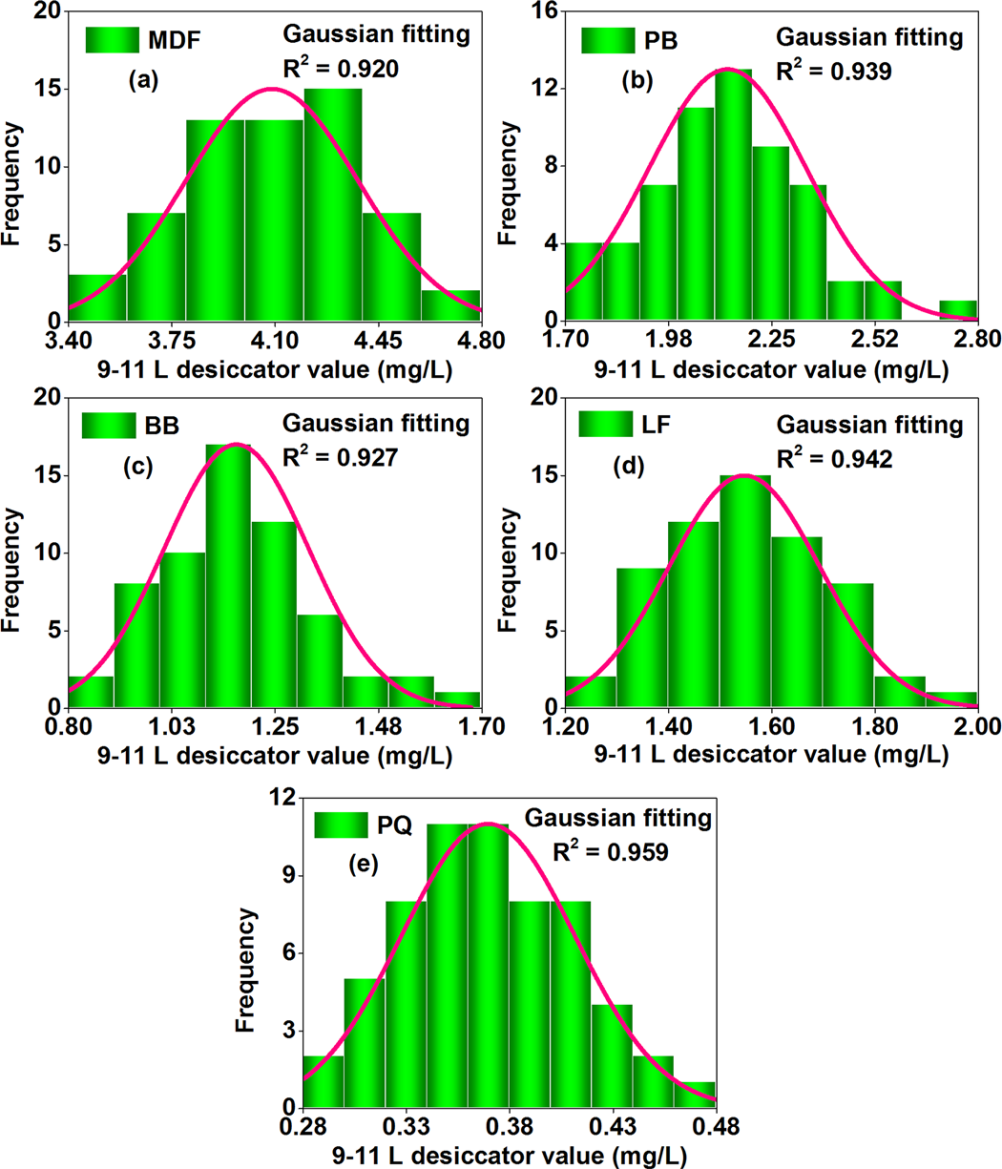
Statistical distributions of formaldehyde emission values (9–11 L desiccator value, derived from multiple tests) of various materials characterized by the 9–11 L desiccator method of the Chinese national standard GB 18580. (a) MDF, medium density fiberboard. (b) PB, particleboard. (c) BB, blockboard. (d) LF, laminate flooring. (e) PQ, parquet. The frequency denoted the number of data occurred at corresponding emission formaldehyde levels. These data were fitted by the probability density function of a Gaussian distribution.

**Fig 10:**
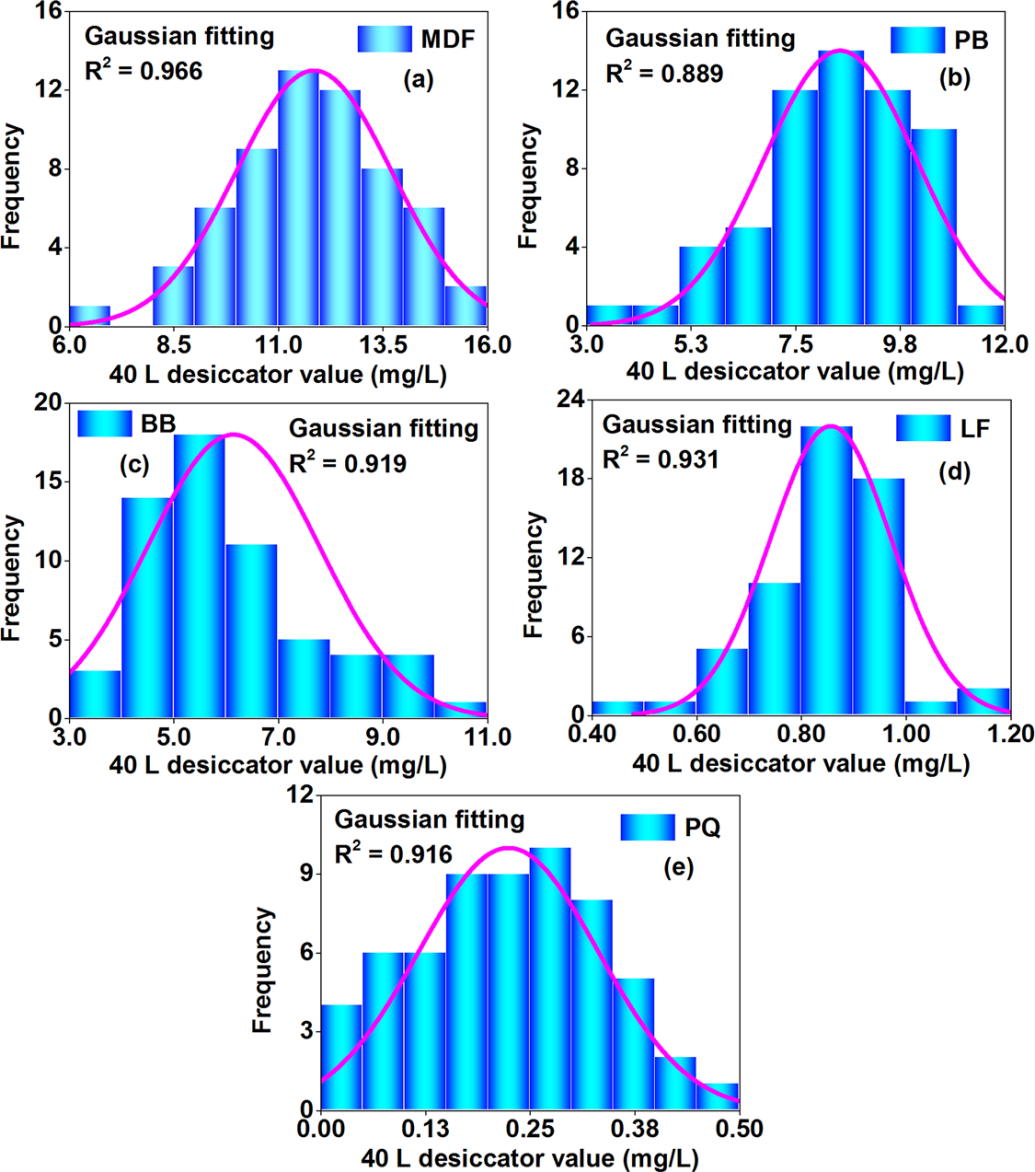
Statistical distributions of formaldehyde emission values (40 L desiccator value, derived from multiple tests) of various materials characterized by the 40 L desiccator method of the Chinese national standard GB 18580. (a) MDF, medium density fiberboard. (b) PB, particleboard. (c) BB, blockboard. (d) LF, laminate flooring. (e) PQ, parquet. The frequency denoted the number of data occurred at corresponding formaldehyde emission levels. These data were fitted by the probability density function of a Gaussian distribution.

As illustrated in Figs 8–10, in the perforator extraction method, the 9–11 L desiccator method, and the 40 L desiccator method of the Chinese national standard GB 18580, formaldehyde emission values for each wood building material (of the 5 materials) were distributed unequally among multiple tests, which were all shaped like a “bell curve” with a few results at the lower end (the low formaldehyde emission value), a few results at the upper end (the high formaldehyde emission value), and most results clumped in the middle (the medium formaldehyde emission value). Considering the similarity to a Gaussian distribution (or a normal distribution), data of each material-method combination (of the15 combinations = 5 materials × 3 methods) were respectively fitted with a probability density function like Eq (2), whose R^2^ were all over 0.81, thus providing a good indication of adequate model performance according to the American ASTM D 5157 [38]. De facto, the Gaussian distribution is very common in the research of wood composites; for instance, some physical and mechanical properties of wood products are recently demonstrated to be normally distributed, such as the compressive strength of fiber-reinforced composites and, the density and the ultimate strength of scrimbers, which further indicates the plausibility of a Gaussian distribution for formaldehyde emission values of wood building materials in this study [56,57].

$$f=\beta +\frac{\alpha }{\sigma \sqrt{2\pi }}\exp\left[-\frac{{\left(x-\mu \right)}^{2}}{2\sigma^{2}}\right]$$ (2)

where *f* is the probability density function of a Gaussian distribution, *x* is the random variable (the formaldehyde emission value characterized by the perforator extraction method, the 9–11 L desiccator method, or the 40 L desiccator method), *μ* and *σ* are respectively the arithmetic mean and the standard deviation of the random variable *x*, *α* and *β* are parameters for this model.

**Fig 11:**
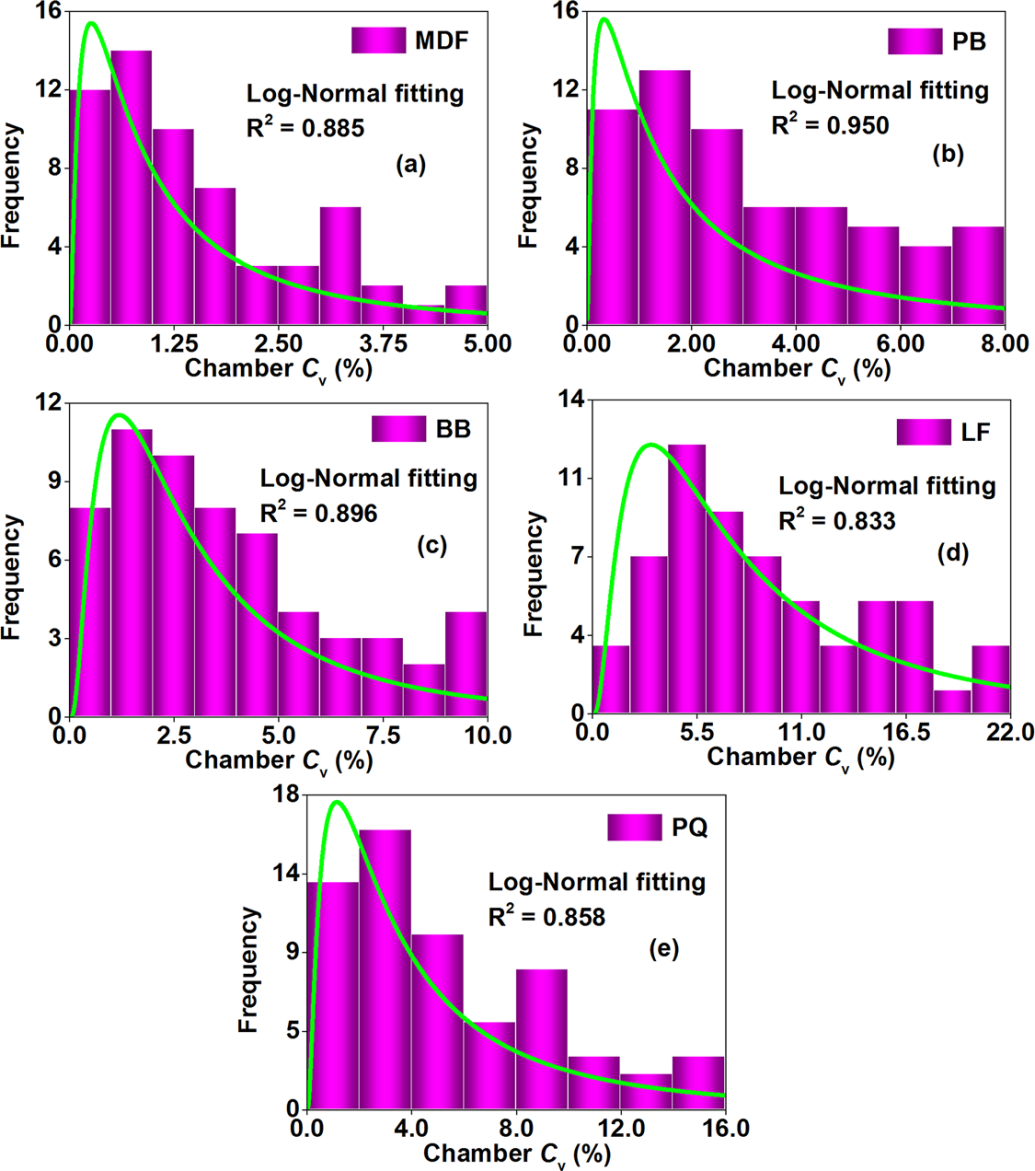
Statistical distributions of formaldehyde emission data (chamber *C*_v_, derived from multiple tests) of various materials characterized by the environmental chamber method of Chinese national standard GB 18580. Chamber *C*_v_, the coefficient of variation for each formaldehyde emission concentration during the environmental chamber test caused by the duplicate sampling. (a) MDF, medium density fiberboard. (b) PB, particleboard. (c) BB, blockboard. (d) LF, laminate flooring. (e) PQ, parquet. The frequency denoted the number of data occurred at corresponding chamber *C*_v_ levels. These data were fitted by the probability density function of a log-normal distribution.

As depicted in Fig 11, for the environmental chamber method of the Chinese national standard GB 18580, formaldehyde emission data (chamber *C*_v_, the coefficient of variation for each formaldehyde emission concentration during the environmental chamber test caused by the duplicate sampling) for each wood building material (of the 5 materials) were also distributed unequally among multiple tests; but unlike a “bell curve” for the perforator extraction method, the 9–11 L desiccator method, and the 40 L desiccator method, it featured a few results at the upper end (the high variation level for the formaldehyde emission concentration), and most results clumped at the lower end (the low variation level for the formaldehyde emission concentration). Considering the comparability to a log-normal distribution, data of each material (of the 5 materials) were fitted with a probability density function like Eq (3), whose R^2^ were all beyond 0.81, thus providing a good indication of adequate model performance according to the American ASTM D 5157 [38]. In fact, the log-normal distribution is also very common in the research of wood composites; for example, some data with regard to the indoor application of wood products are recently confirmed to be log-normally distributed, such as the formaldehyde emission concentration for newly prefabricated houses and, the area and the (wood products) loading factor for general Chinese bedrooms and living rooms, which further reflects the soundness of a log-normal distribution for formaldehyde emission data of wood building materials in this study [48,58].

$$f=\beta +\frac{\alpha }{\sigma_{\text{log}}\sqrt{2\pi }\cdot x}\exp\left[-\frac{{\left(\ln x-\mu_{\text{log}}\right)}^{2}}{2\sigma_{\text{log}}{}^{2}}\right]$$ (3)

where *f* is the probability density function of a log-normal distribution, *x* is the random variable (chamber *C*_v_, the coefficient of variation for each formaldehyde emission concentration during the environmental chamber test caused by the duplicate sampling), *μ*_log_ and *σ*_log_ are respectively the natural logarithmic mean and the natural logarithmic standard deviation of the random variable *x*, *α* and *β* are parameters for this model.

In terms of the probability theory, the natural logarithm of random variables subject to a log-normal distribution would comply with a Gaussian distribution [16]. Similar to the arithmetic mean for a Gaussian distribution, the most probable value for a log-normal distribution is generally the geometric mean [58].

#### Step 2 of uncertainty evaluation: A large scale simulation survey

In terms of statistics, in case of a smaller “sample” size (*i*.*e*., the limited number of formaldehyde emission data in this research) and a unknown standard deviation of corresponding “population”, the Student’ s *t*-distribution can be employed to determine the measurement uncertainty of the arithmetic mean for a Gaussian distribution, or a Gaussian distribution caused by the natural logarithm of random variables subject to a log-normal distribution [16]. For a Gaussian distribution like Eq (4), the above conclusion can be expressed as Eq (5).

x~N(μ,σ) (4)

$$\frac{m-\mu }{s/\sqrt{n}}∼t(n-1)$$ (5)

where *x* is the random variable (*i*.*e*., “sample”, formaldehyde emission data in this research), *n* is the “sample” size (the number of formaldehyde emission data in this research for each material-method combination of the 20 combinations = 5 material × 4 methods), *m* and *s* are respectively the arithmetic mean and the standard deviation of the random variable *x*, *μ* and *σ* are respectively the arithmetic mean and the standard deviation of the approximate “population” produced by a large scale simulation survey based on the “sample”.

Then, by virtue of the MATLAB 7.0 (a popular language of technical computing, developed by Mathworks, USA), about 60,000 random variable *p*_*i*_ values (*i* = 1 to 60,000) subject to a Student’ s *t*-distribution were generated (the rationality of the simulation times 60,000 chosen in this study was discussed in “Step 3 of uncertainty evaluation”), which corresponded to the *t*(*n*-1) in Eq (5), thus updating Eq (5) into Eq (6):

$$\mu_{i}=m-p_{i}\frac{s}{\sqrt{n}}$$ (6)

**Fig 12:**
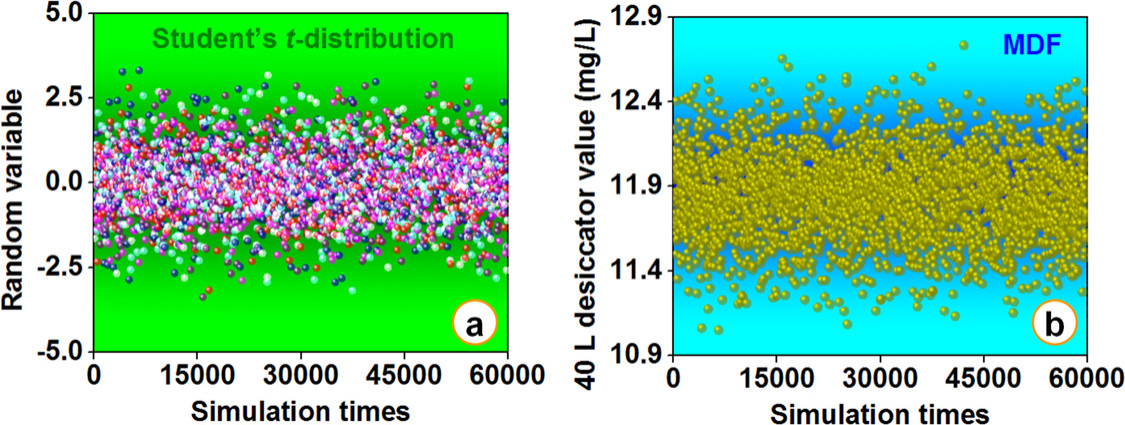
A large scale simulation survey for formaldehyde emissions of various materials in standard tests of the Chinese national standard GB 18580. (a) The random variable *p*_*i*_ values (*i* = 1 to 60,000) subject to the Student’s *t*-distribution generated by the computer. (b) An illustrative example of the large scale simulation survey, *i*.*e*., the simulated *μ*_*i*_ values (*i* = 1 to 60,000) of the 40 L desiccator test for the medium density fiberboard (MDF), in which the *μ*_*i*_ values (*i* = 1 to 60,000) represented the formaldehyde emission value characterized by the 40 L desiccator method.

For each material-method combination (of the 20 combinations = 5 materials × 4 tests) in this research, combining the random variable *p*_*i*_ values (*i* = 1 to 60,000) and Eq (6), the corresponding 60,000 simulated formaldehyde emission data *μ*_*i*_ values (*i* = 1 to 60,000) can be obtained, which was equal to launching 60,000 formaldehyde emission tests for each material-method combination, thus providing a data resource for the uncertainty evaluation in this work [16]. As exhibited in Fig 12, most random variable *p*_*i*_ values (*i* = 1 to 60,000) subject to the Student’ s *t*-distribution generated by the computer were in the range of -2.5 to 2.5; while, as an illustrative example, the simulated *μ*_*i*_ values (*i* = 1 to 60,000) of the 40 L desiccator test for the fiberboard were overall in the range of 11.4 mg/L to 12.4 mg/L, in which the *μ*_*i*_ value (*i* = 1 to 60,000) denoted the formaldehyde emission value characterized by the 40 L desiccator method.

#### Step 3 of uncertainty evaluation: Uncertainty definition and assessment

In this study, the measurement uncertainty in Chinese standard tests for product formaldehyde emissions was calculated according to Eq (7), which can be considered a ratio of the deviation to the mean of data. To analyze the effect of the simulation times on the measurement uncertainty evaluation, the results were obtained according to Eq (7) but under 3 situations: the simulation times *N =* 600 (*i*.*e*., *μ*_1_ to *μ*_600_ were used to estimated the uncertainty), the simulation times *N =* 6,000 (*i*.*e*., *μ*_1_ to *μ*_6,000_ were used to estimated the uncertainty), and the simulation times *N =* 60,000 (*i*.*e*., *μ*_1_ to *μ*_60,000_ were used to estimated the uncertainty).

$$U=\{\begin{array}{c} \frac{S}{M}\times 100\%\\ {\left[\prod_{i=1}^{N}\exp(\mu_{i})\right]}^{1/N} \end{array}\begin{array}{c} ,\\ , \end{array}\begin{array}{c} \begin{array}{l} \text{for the perforator extraction and desiccator tests},\\ \text{whose data comply with a Gaussian distribution}; \end{array}\\ \begin{array}{l} \;\text{for the environmental chamber test},\\ \;\text{whose data comply with a log-normal distribution}. \end{array} \end{array}$$ (7)

where *U* is the measurement uncertainty, *N* is the simulation times, *μ*_*i*_ (*i* = 1 to *N*) is the formaldehyde emission data obtained in the large scale simulation survey (for the perforator extraction method, the 9–11 L desiccator method, and the 40 L desiccator method, the *μ*_*i*_ value (*i* = 1 to *N*) denotes the formaldehyde emission value; while for the environmental chamber method, the *μ*_*i*_ value (*i* = 1 to *N*) represents the natural logarithm of “chamber *C*_v_—the coefficient of variation for each formaldehyde emission concentration during the environmental chamber test caused by the duplicate sampling”), *M* and *S* are respectively the arithmetic mean and the standard deviation of the *μ*_*i*_ values (*i* = 1 to *N*). As seen from Eq (7), for the perforator extraction method, the 9–11 L desiccator method, and the 40 L desiccator method, the measurement uncertainty can be considered a ratio of “the standard deviation of formaldehyde emission values from multiple tests” to “the arithmetic mean of formaldehyde emission values from multiple tests”; while, for the environmental chamber method, the measurement uncertainty can be considered a geometric mean of multiple chamber *C*_v_.

**Fig 13:**
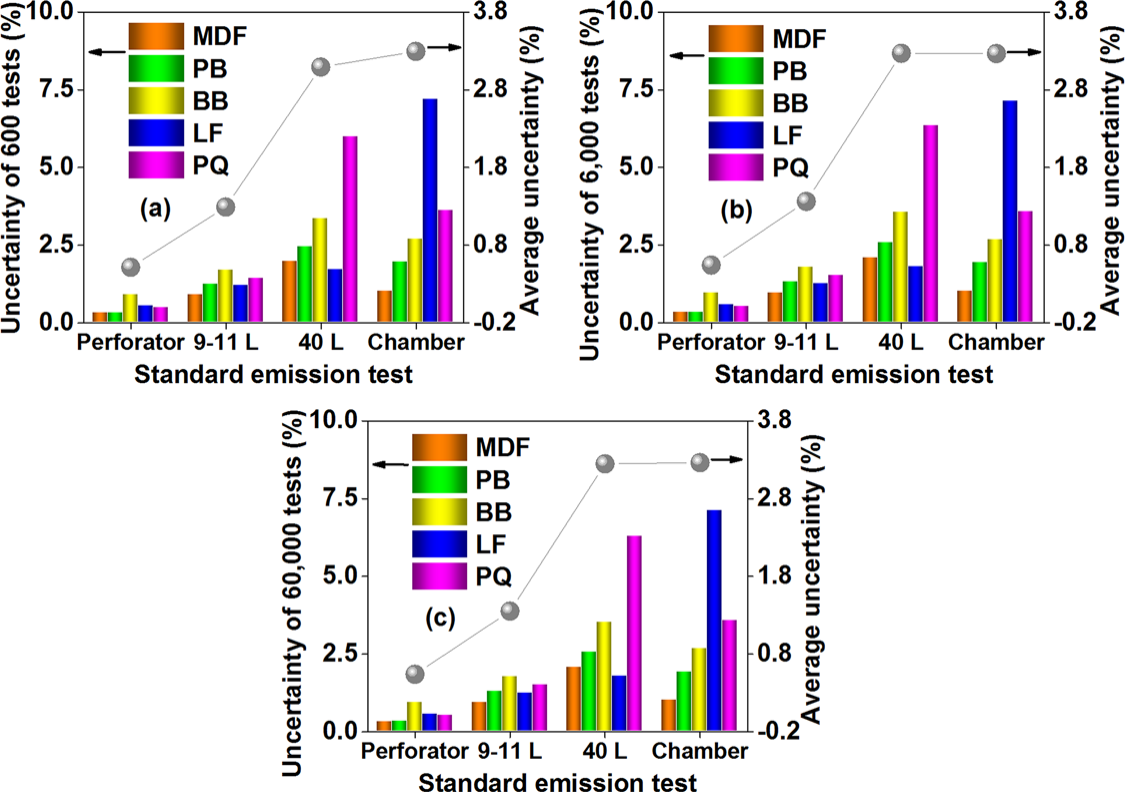
The measurement uncertainty for formaldehyde emission data of various materials characterized by test methods of the Chinese national standard GB 18580, which is evaluated by the Monte Carlo method. (a) The simulation times *N* of the large scale simulation survey = 600. (b) The simulation times *N* of the large scale simulation survey = 6,000. (c) The simulation times *N* of the large scale simulation survey = 60,000. MDF, medium density fiberboard; PB, particleboard; BB, blockboard; LF, laminate flooring; PQ, parquet. Perforator, the perforator extraction method; 9–11 L, the 9–11 L desiccator method; 40 L, the 40 L desiccator method; Chamber, the environmental chamber method. The “uncertainty” denoted the measurement uncertainty for a material-method combination (of the 20 combinations = 5 materials × 4 methods), while the “average uncertainty” represented the measurement uncertainty for a method (of the 4 methods), which was the arithmetic mean of the 5 “uncertainties” for the the 5 materials studied by this method.

As illustrated in Fig 13, between the 3 simulation times (*N* = 600, *N* = 6,000, and *N* = 60,000), the estimated measurement uncertainty of various material-method combinations (of the 20 combinations = 5 materials × 4 tests) changed slightly, which indicated that the simulation times chosen in this study would be appropriate for performing a measurement uncertainty evaluation on Chinese standard tests for product formaldehyde emissions. On the other hand, the measurement uncertainty would be influenced by some material and test factors.

For the effect of material factors, the measurement uncertainty seemed to rise when the structure of wood composites became more complicated (*e*.*g*., from wood based panels to finishing products, or from the similar structure and density to the anisotropy) [48]. For example, uncertainties of the fiberboard and particleboard (typical wood based panels) were relatively low and stable in various tests, but uncertainties of the blockboard, flooring, and parquet (typical finishing products) showed higher levels and greater variations in various tests. Considering that the formaldehyde emission behavior of wood building materials features an internal diffusion-controlled mechanism that is closely connected with the material structure, the effect of material factors may be understandable [45]. Likewise, in an inter-laboratory comparison for the formaldehyde emission value of the particleboard (wood based panels) and the flooring (finishing products) using the environmental chamber method, only the results of the flooring show highly significant differences between the laboratories, thus highlighting the greater uncertainty caused by a more complicated material structure [59].

For the effect of test factors, the measurement uncertainty seemed to rise with the increasing “scale factor” (“scale factor”, *e*.*g*., the dimension of the test specimen, the volume of the test container, or the duration of the test procedure). For instance, the average uncertainty (for the 5 materials) of the perforator extraction method was about 0.5%, but that of the environmental chamber method exceeded 3%. Considering that experimental conditions in a smaller scale test is more convenient to be controlled, the effect of test factors may be understandable [54]. Moreover, the average uncertainty (for the 5 materials) of the 40 L desiccator method was very close to that of the environmental chamber method, thus implying their higher similarity again, which (the higher similarity between 40 L desiccator and chamber tests) also agreed with the results and discussion in the section of “Product emission levels in Chinese standard tests” (see Fig 4, and the corresponding discussion).

By and large, the measurement uncertainty in Chinese standard tests for product formaldehyde emissions was overall low, in which uncertainties for the 20 material-method combinations (= 5 materials × 4 methods) were all below 7.5%, while the average uncertainty for each test (of the 4 standard emission tests) was under 3.5%, thus being acceptable in the engineering application. In this sense, Chinese standard test methods can be employed to properly characterize the formaldehyde emissions of wood building materials.

### Data correlations between Chinese standard tests

#### Available data correlations between some standard tests

To compare and understand formaldehyde emission data of wood building materials characterized by different standard test methods, the study on their empirical correlations has received widespread attentions. As presented in Table 5, the linear model is commonly used to correlate formaldehyde emission data [50–54,63–66,69–71]. On one hand, a positive correlation (the slope > 0) for formaldehyde emission data between various standard test methods can be observed in a linear relation, while the effect of material and test factors on data correlations seems relatively complicated; for example, a high R^2^ can be caused by many kinds of data correlations, but in different reports, data correlations of similar materials and tests can also lead to the greatly different R^2^ [63,64]; overall, the linear model often harvests a higher R^2^ (≥ 0.81) in previous work. On the other hand, as an important parameter, the slope of a linear model plays a key role when converting and comparing formaldehyde emission data between different standard tests, whose value would be affected by the definition of the *y* method (the dependent variable) and the *x* method (the independent variable) in the relationships [63]; for instance, the data correlations (expressed as the dependent variable *vs*. the independent variable in the relationships) such as the perforator method *vs*. the desiccator method, the perforator method *vs*. the chamber method, and the desiccator method *vs*. the chamber method always give slopes > 1 [50,54,63,65], which indicates that the formaldehyde emission value characterized by the *y* method (the dependent variable) would usually be numerically larger than that characterized by the *x* method (the independent variable), and may be attributed to inherent differences in the physical meaning of formaldehyde emission values between various standard test methods [38]. Accordingly, once the *y* method (the dependent variable) and the *x* method (the independent variable) are exchanged in the relationships, these correlations would yield slopes < 1 [50,54,69,70].

**Table 5:** Reported Data Correlations for Product Formaldehyde Emissions between Some Standard Test Methods of Developed Countries and Regions.

Data correlations for the perforator method, the desiccator method, and the chamber test				
Material	Method *y vs*. *x*	Mathematical Model	R^2^	Reference
MDF	PE *vs*. DP2	*y* = 1.4406 *x* + 2.5706	0.9756	[50]
MDF	DP2 *vs*. PE	*y* = 0.6772 *x* - 1.6795	0.9756	[50]
PB	PE *vs*. CAD	*y* = 22.79 *x* + 1.92	0.93	[54]
PB	CE1 *vs*. PE	*y* = 0.04 *x* - 0.07	0.88	[54]
PB	CE1 *vs*. CAD	*y* = 0.95 *x* - 0.008	0.94	[54]
MDF	PE *vs*. DK	*y* = 8.6407 *x* + 0.5315	0.967	[63]
MDF	CI *vs*. DK	*y* = 0.0882 *x* + 0.0710	0.752	[63]
PB	PE *vs*. DK	*y* = 4.9307 *x* + 1.3773	0.920	[63]
PB	CI *vs*. DK	*y* = 0.1078 *x* + 0.0463	0.839	[63]
PW	CI *vs*. DK	*y* = 0.0252 *x* + 0.0980	0.921	[63]
PB	DJ *vs*. PE	Not given	0.7099	[64]
PB	DJ *vs*. CE1	Not given	0.7204	[64]
PQ	DP1 *vs*. CS1	*y* = 8.8206 *x* + 0.0711	0.6183	[65]
MDF and PB	CAE2 *vs*. DJ	*y* = (0.115 to 0.471) *x* + (- 0.129 to 0.007)	0.860 to 0.995	[69]
PB	CAE1 *vs*. DA	*y* = (0.030 to 0.332) *x* + (- 0.119 to 0.031)	0.902 to 0.999	[70]
PB	CAE2 *vs*. DJ	*y* = 0.76 *x* - 0.025	0.97	[71]
PW	CAE2 *vs*. DJ	*y* = 0.166 *x* - 0.023	0.99	[71]
Data correlations involving other methods				
Material	Method *y vs*. *x*	Mathematical Model	R^2^	Reference
PQ	FLEC *vs*. DJ	*y* = 0.262 *x* - 0.162	0.92	[51]
PQ	CK *vs*. DJ	*y* = 0.099 *x* + 0.024	0.97	[51]
MDF	GA *vs*. PE	*y* = 0.38 *x - *0.96	0.77	[52]
PB	GA *vs*. PE	*y* = 0.15 *x - *0.23	0.93	[52]
SW	CE2 *vs*. GA	*y* = (0.098 to 0.36) *x* + (- 0.0142 to 0.003)	0.53 to 0.94	[53]
PB and LF	GA *vs*. PE	*y* = 0.09 *x* + 0.03	0.90	[54]
PB and LF	GA *vs*. CAD	*y* = 2.15 *x* + 0.20	0.89	[54]
PB and LF	CE1 *vs*. GA	*y* = 0.41 *x* - 0.08	0.90	[54]
PB and LF	PE *vs*. GA	Cubic polynomials	0.65 to 0.82	[55]
MDF	DL *vs*. CS2	*y* = 1.0475 *x* + 0.0006	0.8429	[65]
MDF	FLEC *vs*. CS1	*y* = 0.6916 *x* + 0.0193	0.8681	[65]
PQ	DL *vs*. CS2	*y* = 1.2384 *x - *0.0169	0.9156	[65]
PQ	FM *vs*. CS1	*y* = 39.816 *x - *0.7904	0.5504	[65]
MDF	CS1 *vs*. FLEC	*y* = 1.1954 *x - *0.0071	0.9023	[66]

Wood building materials

(a) MDF, medium density fiberboard

(b) PB, particleboard

(c) LF, laminate flooring

(d) PQ, parquet

(e) PW, plywood

(f) SW, solid wood.

The result of the perforator method

(a) PE (mg/100g), the perforator extraction method of the European EN 120.

The result of the desiccator method

(a) DA (mg/L), the 9–11 L desiccator method of the American ASTM D 5582

(b) DJ (mg/L), the 9–11 L desiccator of the Japanese JIS A 1460

(c) DP1 (mg/L) and DP2 (ppm), the 9–11 L desiccator of the Japanese JIS A 5908

(d) DK (mg/L), the 9–11 L desiccator method of the Korean KS M 1998–4.

The result of the chamber method

(a) CI (mg/m^3^), the 1 m^3^ chamber of the International ISO/FDIS 12460–1

(b) CE1 (mg/m^3^) and CE2 (ppm), the 0.225 m^3^ chamber method of the European EN 717–1

(c) CS1 (mg/m^3^) and CS2 (ppm), the 1 m^3^ chamber of the Swedish SS 27 02 36

(d) CAD (ppm), the 1 m^3^ chamber method of the American ASTM D 6607–02

(e) CAE1 (mg/m^3^) and CAE2 (ppm), the 28 m^3^ chamber method of the American ASTM E 1333

(f) CK (mg/m^2^ h), the 20 L chamber method of the Korean KS M 1998–2.

The result of other methods

(a) FLEC (mg/m^2^ h), the field and laboratory emission cell method of the European ENV 13419–2

(b) GA (mg/m^2^ h), the gas analysis method of the European EN 717–2

(c) FM (mg/kg), the flask method of the European EN 717–3

(d) DL (ppm), the desiccator lid method of Swedish Casco Co., Ltd.

Since a linear model may not work so well in some situations [63–65], polynomials have been occasionally employed to improve the R^2^ of data correlations; but compared to the linear model, the polynomial model still lacks a stable form [52,55]. Considering the difficulty in finding a general model, the scope of application for various models should be carefully recognized, which would contribute to harvesting better data correlations. For example, a R^2^ of the data correlation between the chamber method and the desiccator method can be improved when the chamber test value is obtained at a lower loading factor (in the range of 0.13 to 0.04 ft^2^/ft^3^) or a lower air change rate (in the range of 1.0 to 0.5 h^-1^); however, this modification may fail once the loading factor drops to 0.02 ft^2^/ft^3^, thus reflecting the scope of application of a linear model for this case [69]. Similarly, there would be many factors in details and procedures of formaldehyde emission tests (*e*.*g*., the product type, the exposed edge of the specimen, and the conditioning period) that affect the data correlations, which should be taken seriously if necessary [59,70].

#### Modeling and validation for Chinese standard tests

**Fig 14:**
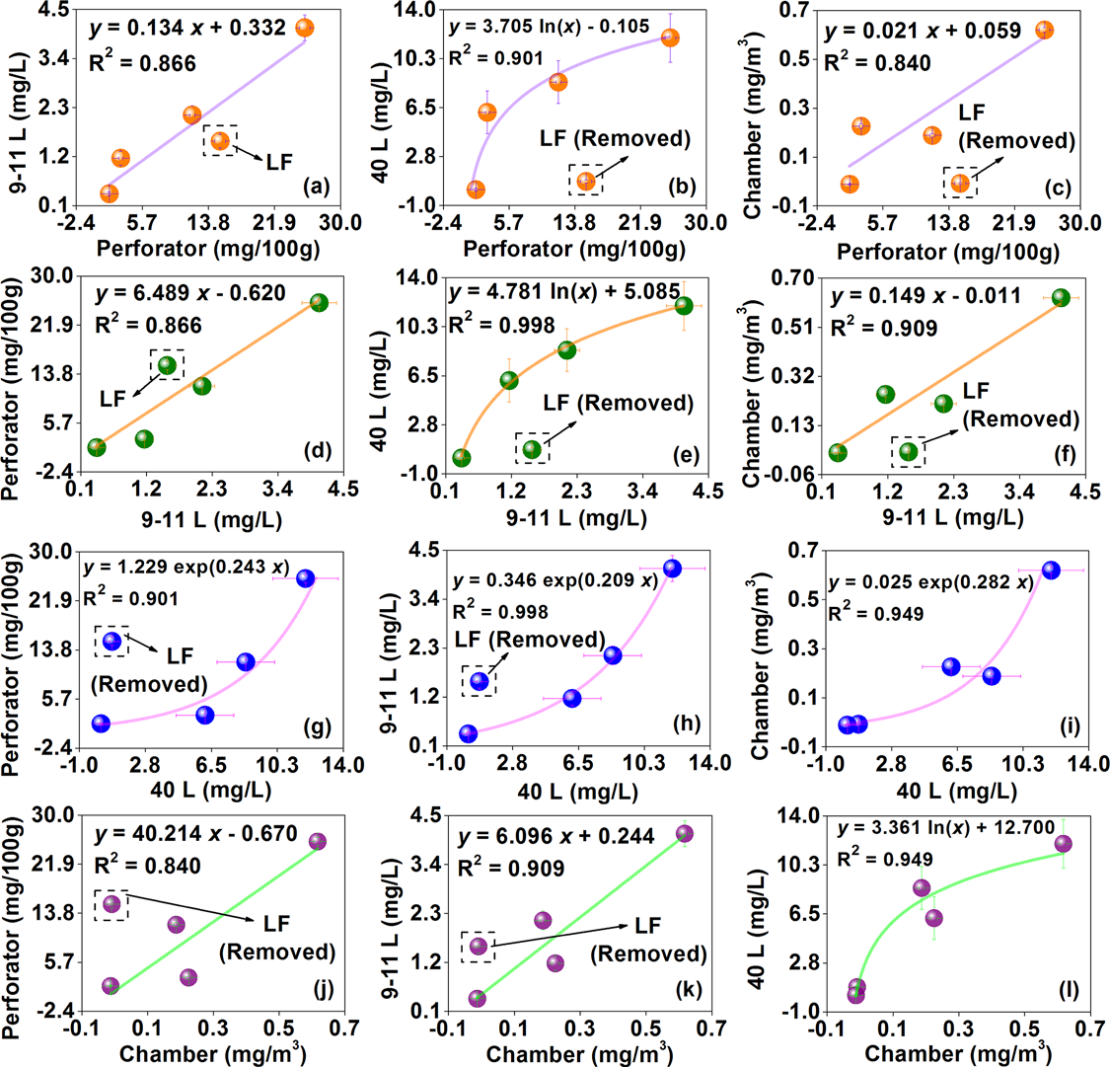
Empirical correlations for formaldehyde emission values of various materials characterized by different test methods of the Chinese national standard GB 18580. Perforator, the results of the perforator extraction method; 9–11 L, the results of the 9–11 L desiccator method; 40 L, the results of the 40 L desiccator method; Chamber, the results of the environmental chamber method. LF, laminate flooring. Considering that data of the laminate flooring occasionally served as an outlier, when the correlation was obviously affected by this problem, data of the laminate flooring would be marked as “LF” in corresponding Figs. When data of the laminate flooring were marked as “Removed”, in corresponding Figs, they would not be considered when modeling.

As illustrated in Fig 14, 2 mathematical models were successfully applied in this study to correlate formaldehyde emission values of various wood building materials characterized by different test methods of the Chinese national standard GB 18580, whose R^2^ were all over 0.81, thus providing a good indication of adequate model performance according to ASTM D 5157 [38]. Moreover, the results in this study can provide a basis for the further comparison of standard test methods between China and developed countries and regions in the future research, thus promoting the technology exchange and collaboration around the world.

For one thing, a linear model was overall applicable to correlate the formaldehyde emission value between the Chinese perforator extraction method, 9–11 L desiccator method, and environmental chamber method. For example, the R^2^ of the data correlation between the perforator extraction method and the 9–11 L desiccator method can be 0.866 (Fig 14(A) and Fig 14(D)), which indicated the scope of application for a linear model in this research. But when considering all the 5 wood building materials, the R^2^ of the data correlation between the perforator extraction method and the environmental chamber method (Fig 14(C) and Fig 14(J)), and that between the 9–11 L desiccator method and the environmental chamber method (Fig 14(F) and Fig 14(K)) were only 0.485 and 0.807 respectively, in which the formaldehyde emission value of the flooring seemed to serve as an outlier. After omitting data of the flooring, the 2 lower R^2^ respectively rose to 0.840 and 0.909, while this modification can also lead to an improved R^2^ (from 0.866 to 0.977) for the data correlation between the perforator extraction method and the 9–11 L desiccator method, thus further indicating the scope of application for a linear model in some specific situations of this research. Besides, the data correlations (expressed as the dependent variable *vs*. the independent variable in the relationships) of the perforator extraction method *vs*. the 9–11 L desiccator method (Fig 14(D)), the perforator extraction method *vs*. the environmental chamber method (Fig 14(J)), and the 9–11 L desiccator method *vs*. the environmental chamber method (Fig 14(K)) all gave slopes > 1, which agreed with previous reports using corresponding standard test methods (the perforator, desiccator, chamber methods) of developed countries and regions [50,54,63,65].

For another, a logarithmic (or exponential) model can work better for data correlations involving the 40 L desiccator method (expressed as the dependent variable *vs*. the independent variable in the relationships, a logarithmic model applied to data correlations of the 40 L desiccator method *vs*. other 3 methods, while an exponential model applied to data correlations of other 3 methods *vs*. the 40 L desiccator method). For instance, the R^2^ of the data correlation between the 40 L desiccator method and the environmental chamber method can be up to 0.949 (Fig 14(I) and Fig 14(L)), which indicated the scope of application for a logarithmic (or exponential) model in this research. But when considering all the 5 materials, the formaldehyde emission value of the flooring also seemed to serve as an outlier that compromised the data correlation between the 40 L desiccator method and the perforator extraction method (Fig 14(B) and Fig 14(G)), and that between the 40 L desiccator method and the 9–11 L desiccator method (Fig 14(E) and Fig 14(H)), which was partly similar to the situation of applying a linear model on data correlations of other 3 methods. After omitting data of the flooring, the 2 lower R^2^ climbed respectively from 0.335 to 0.901, and from 0.676 to 0.998, thus further indicating the scope of application for a logarithmic (exponential) model in some specific situations of this research. De facto, the data correlation between the 40 L desiccator method and the environmental chamber method was never remarkably affected by the product type (in other words, this data correlation was applicable to all the 5 materials), thus implying their higher similarity again, which (the higher similarity between 40 L desiccator and chamber tests) also coincided with the results and discussion in the sections of “Product emission levels in Chinese standard test methods” (see Fig 4, and the corresponding discussion) and “Measurement uncertainties in Chinese standard test method” (see Fig 13, and the corresponding discussion).

Based on the above discussion, data correlations between various test methods of the Chinese national standard GB 18580 can be combined to constitute an integrated model as Eq (8):

$$y=\{\begin{array}{c} \alpha \cdot x+\beta \\ \alpha \cdot \ln(x)+\beta \\ \beta \cdot \exp(\alpha \cdot x) \end{array}\begin{array}{c} ,\\ ,\\ , \end{array}\begin{array}{c} \text{among perforator},\;9\text{-}11\;\text{L desiccator},\;\text{chamber methods}\\ \;y=\text{the}\;40\;\text{L desiccator method},\;x=\text{the other}\;3\;\text{methods}\\ \;y=\text{the other}\;3\;\text{methods},\;x=\text{the}\;40\;\text{L desiccator methods} \end{array}$$ (8)

where *y* (the dependent variable) and *x* (the independent variable) are respectively the formaldehyde emission values of the same material characterized by different Chinese standard test methods, *α* and *β* are parameters for this model.

In previous work, a logarithmic (exponential) model has been barely reported, probably because this relationship is hidden in the Chinese 40 L desiccator method. By virtue of this model, formaldehyde emission data of the 40 L desiccator method can be well correlated with those of other 3 methods; especially the data correlation of the 40 L desiccator method with the environmental chamber method, it was valid for all the 5 wood building materials in this research. In this sense, the Chinese 40 L desiccator method may serve as a feasible alternative to the environmental chamber method in engineering application, which can not only achieve the reliable and accurate results but also solve the problems of the higher device cost (*e*.*g*., constructing the larger chamber) and the longer test time (*i*.*e*., 7 to 28 days) in current environmental chamber methods, thus being very meaningful to the production control and the risk assessment for product formaldehyde emissions.

## Conclusions

In this work, formaldehyde emission characteristics of wood building materials in Chinese standard tests were investigated, which would be the first systematic effort on this issue, and the conclusions were as follows:

1. Formaldehyde emissions of 5 common materials (fiberboard, particleboard, blockboard, flooring, and parquet) were obtained by 4 test methods (the perforator extraction method, the 9–11 L desiccator method, the 40 L desiccator method, and the environmental chamber method) of Chinese national standard GB 18580, while important features of these standard test methods were also recognized through comparing with existing methods and available reports.
2. In terms of the limit of the Chinese GB 18580, different standard test methods may give slightly different evaluations on the product quality. In the perforator extraction test and the 9–11 L desiccator test, the blockboard and parquet reached the E1 grade for the formaldehyde emission, which can be directly used in the indoor environment; but in the 40 L desiccator test and the environmental chamber test, the flooring and parquet achieved this grade.
3. In multiple tests, formaldehyde emission data of various materials characterized by the perforator extraction method, the 9–11 L desiccator method, and the 40 L desiccator method (*i*.*e*., formaldehyde emission values for the 3 methods) complied with a Gaussian distribution, while those characterized by the environmental chamber method (*i*.*e*., chamber *C*_v_ for this method, the coefficient of variation for each formaldehyde emission concentration during the environmental chamber test caused by the duplicate sampling) followed a log-normal distribution.
4. Based on distributions of formaldehyde emission data, the measurement uncertainty in Chinese standard tests was evaluated by the Monte Carlo method, which was overall low. Uncertainties for the 20 material-method combinations (= 5 materials × 4 methods) were all below 7.5%, while the average uncertainty for each test (of the 4 standard emission tests) was under 3.5%, thus being acceptable in the engineering application. Moreover, a more complicated material structure and a larger test scale were prone to a higher uncertainty.
5. Empirical correlations for formaldehyde emission values of various materials characterized by different Chinese standard test methods were established, in which a linear model can apply to the data correlation between the perforator extraction method, the 9–11 L desiccator method, and the environmental chamber method, with the R^2^ all over 0.840.
6. As for the data correlation involving the 40 L desiccator method, a logarithmic (exponential) model can work better, with the R^2^ all beyond 0.901, in which (expressed as the dependent variable *vs*. the independent variable in the relationships) a logarithmic model applied to data correlations of the 40 L desiccator method *vs*. other 3 methods, while an exponential model applied to data correlations of other 3 methods *vs*. the 40 L desiccator method.
7. For all the characterizations in this research (product emission levels, measurement uncertainties, and data correlations between various methods), results for the 40 L desiccator method presented a greater similarity to those for the environmental chamber method that has been universally considered the most reliable and accurate way for evaluating the effect of product formaldehyde emissions on the indoor air quality, thus highlighting the potential of the Chinese 40 L desiccator method as a more practical approach in the production control and the risk assessment.

This research preliminarily confirmed the effectiveness and the reliability of Chinese standard test methods for product formaldehyde emissions, and the future research would further focus on formaldehyde emission characteristics of other materials in these tests, or systematic comparisons of these methods with various standard test methods of developed countries and regions, especially the application of the Chinese 40 L desiccator method and its influential factors.
